# Inhibition of microRNA-33b specifically ameliorates abdominal aortic aneurysm formation via suppression of inflammatory pathways

**DOI:** 10.1038/s41598-022-16017-5

**Published:** 2022-07-14

**Authors:** Tomohiro Yamasaki, Takahiro Horie, Satoshi Koyama, Tetsushi Nakao, Osamu Baba, Masahiro Kimura, Naoya Sowa, Kazuhisa Sakamoto, Kazuhiro Yamazaki, Satoshi Obika, Yuuya Kasahara, Jun Kotera, Kozo Oka, Ryo Fujita, Takashi Sasaki, Akihiro Takemiya, Koji Hasegawa, Kenji Minatoya, Takeshi Kimura, Koh Ono

**Affiliations:** 1 Department of Cardiovascular Medicine, Graduate School of Medicine, Kyoto University, 54 Shogoin-kawahara-cho, Sakyo-ku, Kyoto, 606-8507 Japan; 2 Division of Translational Research, National Hospital Organization, Kyoto Medical Center, 1-1 Fukakusa Mukaihata-cho, Fushimi-ku, Kyoto, 612-8555 Japan; 3 Department of Cardiovascular Surgery, Graduate School of Medicine, Kyoto University, 54 Shogoin-kawahara-cho, Sakyo-ku, Kyoto, 606-8507 Japan; 4 Graduate School of Pharmaceutical Sciences, Osaka University, 1-6 Yamadaoka, Suita-shi, Osaka, 565-0871 Japan; 5 Center for Drug Design Research, National Institutes of Biomedical Innovation, Health and Nutrition, 7-6-8 Saito-Asagi, Ibaraki-shi, Osaka, 567-0085 Japan; 6 Sohyaku. Innovative Research Division, Mitsubishi Tanabe Pharma Corporation, Shonan Health Innovation Park, 2-26-1, Muraoka-Higashi, Fujisawa-shi, Kanagawa, 251-8555 Japan

**Keywords:** Drug discovery, Cardiology, Medical research

## Abstract

Abdominal aortic aneurysm (AAA) is a lethal disease, but no beneficial therapeutic agents have been established to date. Previously, we found that AAA formation is suppressed in microRNA (miR)-33-deficient mice compared with wild-type mice. Mice have only one miR-33, but humans have two miR-33 s, miR-33a and miR-33b. The data so far strongly support that inhibiting miR-33a or miR-33b will be a new strategy to treat AAA. We produced two specific anti-microRNA oligonucleotides (AMOs) that may inhibit miR-33a and miR-33b, respectively. In vitro studies showed that the AMO against miR-33b was more effective; therefore, we examined the in vivo effects of this AMO in a calcium chloride (CaCl_2_)-induced AAA model in humanized miR-33b knock-in mice. In this model, AAA was clearly improved by application of anti-miR-33b. To further elucidate the mechanism, we evaluated AAA 1 week after CaCl_2_ administration to examine the effect of anti-miR-33b. Histological examination revealed that the number of MMP-9-positive macrophages and the level of MCP-1 in the aorta of mice treated with anti-miR-33b was significantly reduced, and the serum lipid profile was improved compared with mice treated with control oligonucleotides. These results support that inhibition of miR-33b is effective in the treatment for AAA.

## Introduction

The majority of aortic aneurysms in adults are essentially asymptomatic, but the risk of rupture increases as the diameter of the aneurysm increases^1^. Of these, abdominal aortic aneurysms (AAA) have a reported incidence of 1.5 to 2 per 1,000 people per year in the United States^2^. Currently, surgical treatment is considered preferable^3^, whereas treatment with various drugs such as beta-blockers^4^, angiotensin-converting enzyme inhibitors^5^, and calcium channel blockers^6^ are being investigated. However, large-scale randomized controlled trials using these drugs have not shown clear effects. Therefore, the development of therapeutic agents that can inhibit the expansion of aortic aneurysms is of great significance.

Using genetically engineered mice, we previously found that the absence of microRNA (miR)-33 suppressed AAA progression via several anti-inflammatory pathways^7^. Therefore, we decided to use these data to develop a new therapeutic agent for aortic aneurysms. Nucleic acid medicine has been attracting attention as a new therapeutic agent for hereditary and refractory diseases that have been difficult to treat^8,9^. In the development of conventional oligonucleotide therapeutics, there have been problems with stability and efficacy in vivo^10^, but advances in modified nucleic acid technology^11^ and Drug Delivery System technology^12^ have changed this situation, and candidates that are highly effective not only after local administration but also after systemic administration are being developed^13^. Oligonucleotide therapeutics are expected to have high specificity and efficacy similar to antibody drugs and can be produced by chemical synthesis similar to small molecule drugs^10^. In fact, oligonucleotides are designed based on the target RNA sequence, and highly effective oligonucleotides can be obtained in a short time^13^.

In rodents, there is only one miR-33 (miR-33a) in the intron of *sterol regulatory element binding transcription factor 2* (*Srebf2*), but in humans, in addition to miR-33a, there is another miR-33 (miR-33b) in the intron of *SREBF1*. In this study, we developed bridged nucleic acid-modified anti-microRNA oligonucleotides (AMOs) that individually target miR-33a and miR-33b, which differ by only two bases. As a result, we showed that it is possible to target aortic aneurysms and large blood vessels and elucidated its mechanism of action in detail using humanized miR-33b knock-in (KI) mice.

## Results

### miR-33b KI mice showed severe calcium chloride-induced AAA formation

**Figure 1:**
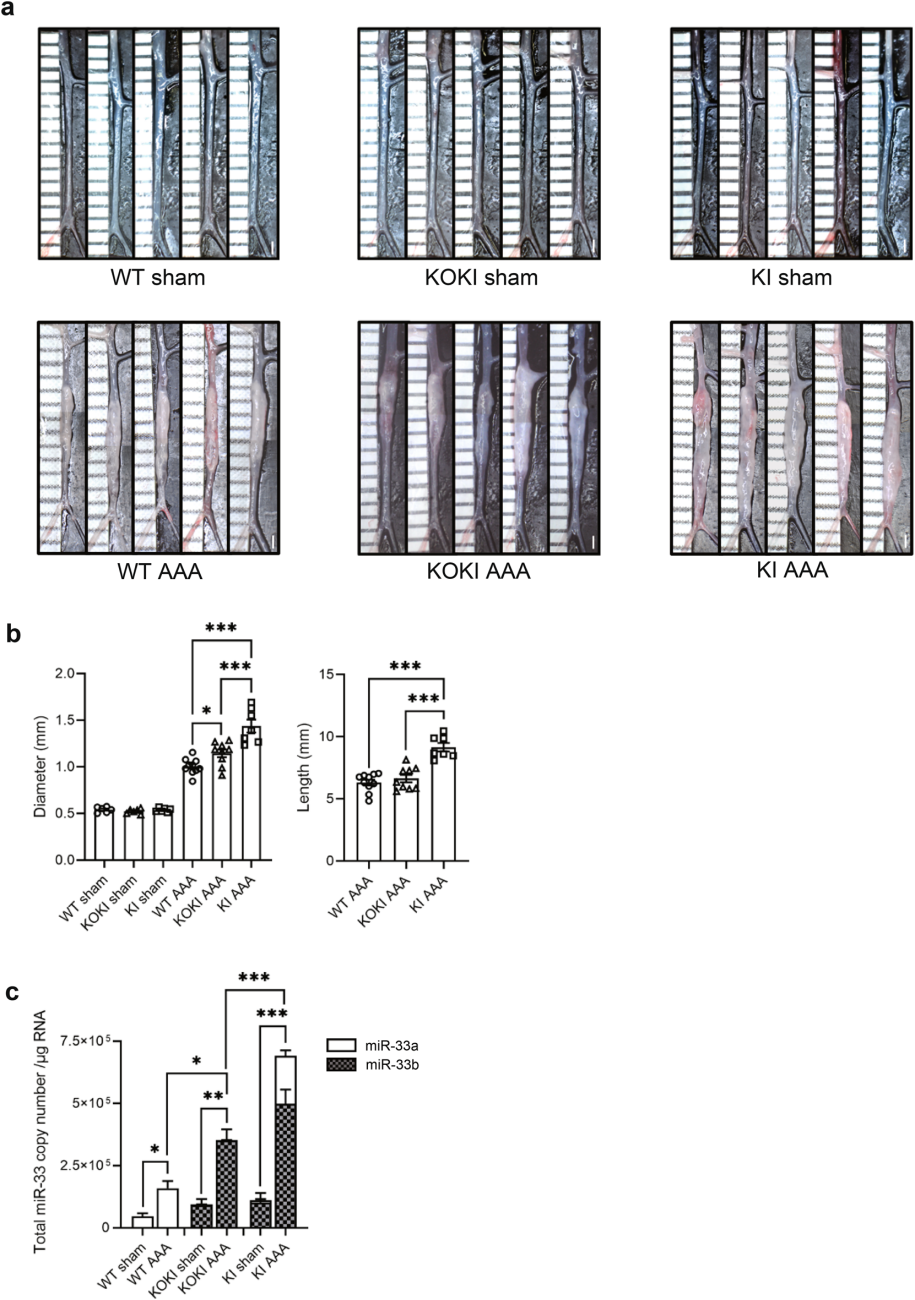
Genetic phenotypes of abdominal aortic aneurysm (AAA) formation in wild-type (WT) mice, miR-33a knock-out and miR-33b knock-in (KOKI) mice, and miR-33b knock-in (KI) mice. (**a**) Representative photographs of sham controls and calcium chloride (CaCl_2_)-induced AAA in WT, KOKI, and KI mice. White bars indicate 1 mm. (**b**) Maximum diameter and lesion length of the abdominal aorta between the left renal artery and the terminal aorta of CaCl_2_-induced AAA, n = 10 mice in WT, n = 9 mice in KOKI, and n = 7 mice in KI mice. One-way ANOVA with Holm–Sidak’s multiple comparisons test. *P < 0.05 and ***P < 0.001. (**c)** Absolute copy numbers of miR-33a and miR-33b in sham (left), CaCl_2_-induced AAA (mid), and integrated graphs, n = 5 mice in each sham, n = 7 mice in WT and KOKI for CaCl_2_-induced AAA, and n = 7 mice in KI for CaCl_2_-induced AAA. One-way ANOVA with Holm–Sidak’s multiple comparisons test (left and mid) and two-way ANOVA with Holm–Sidak’s multiple comparisons test (right). *P < 0.05 and **P < 0.01. All data represent mean ± standard error of the mean (SEM).

Previously, we created a calcium chloride (CaCl_2_)-induced AAA model in miR-33-deficient (miR-33a^−/−^ miR-33b^−/−^) and wild-type (WT) mice (miR-33a^+/+^ miR-33b^−/−^) and found that AAA formation was suppressed in miR-33-deficient mice^7^. This indicated that the amount of miR-33 is related to the worsening of AAA. In the present study, we investigated the effects of miR-33a and miR-33b on AAA in order to clarify the pathogenesis in humans. We previously generated miR-33b KI mice (miR-33a^+/+^ miR-33b^+/+^), which have human miR-33b in intron 16 of *Srebf1*^14^. miR-33b KI mice have both miR-33a and miR-33b as in humans. In these mice, miR-33b is physiologically co-expressed with *Srebf1*. We next crossed miR-33a knock-out (KO) mice with miR-33b KI mice to generate mice with only miR-33b (KOKI) (miR-33a^−/−^ miR-33b^+/+^)^15^. Then, we compared the severity of CaCl_2_-induced AAA formation^16^ in the WT, KOKI, and KI mouse lines. As shown in Fig. 1a,b, the diameter of the aorta was significantly increased in KOKI mice and further increased in KI mice compared with WT mice. In addition, the lesion length of the aorta in KI mice was significantly larger than that in KOKI and WT mice. Next, we measured the copy numbers of miR-33a and miR-33b in 1 µg of total RNA of the aorta (Fig. 1c). As expected, only miR-33a was present in WT mice and only miR-33b was present in KOKI mice. miR-33b levels were higher than miR-33a in KI mice. The copy numbers of both miR-33a and miR-33b increased after AAA formation in all mice, but the total copy number of miR-33a and miR-33b was significantly increased in KOKI compared with WT mice and was further increased in KI mice. In other words, the amount of miR-33 was related to the deterioration of AAA in these genetically modified mice.

### Generation and selection of specific AMOs against miR-33a and miR-33b

**Figure 2:**
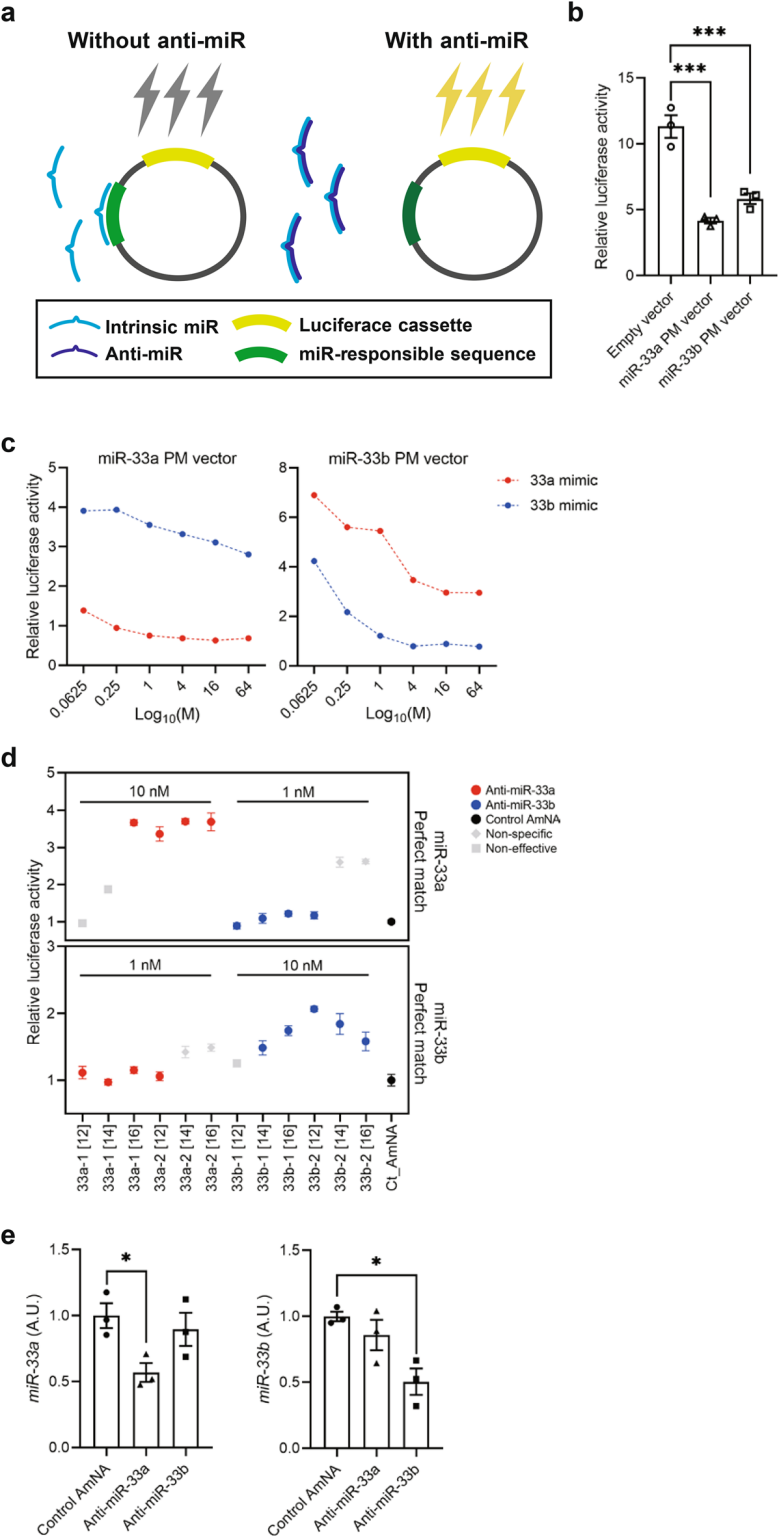

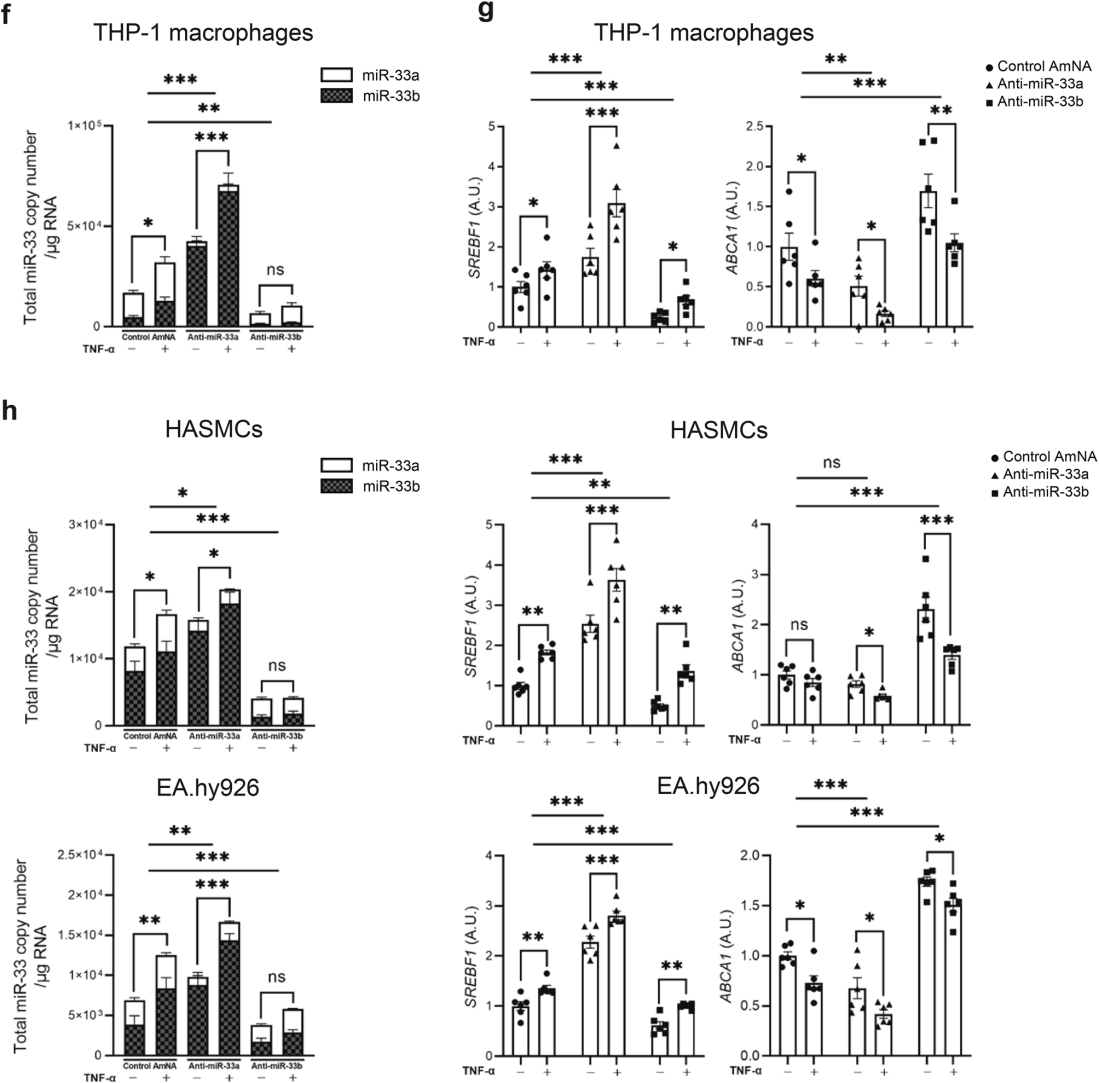
Selection of effective and specific anti-microRNA oligonucleotide (AMOs) against miR-33a and miR-33b. (**a**) Experimental scheme for reporter assay. In the absence of anti-microRNA (miR), intrinsic miR inhibits luciferase expression (left). Adding anti-miR inhibits intrinsic miR, and the expression of luciferase will increase. Luciferase intensity is proportional with inhibition efficiency of anti-miRs (right). (**b**) Reactivities of miR-33a perfect match (33a PM) and miR-33b perfect match (33b PM) reporter vectors in HepG2 cells (human hepatocellular carcinoma cell line), n = 3. One-way analysis of variance (ANOVA) with Dunnett’s multiple comparison test. ***P < 0.001. (**c**) Evaluation of binding specificity of the reporter vectors to miR-33a-mimic (33a mimic) and miR-33b-mimic (33b mimic). (**d**) Screening for candidates of AMOs against miR-33a and miR-33b by efficacy and specificity. (**e**) Expression levels of miR-33a (left, n = 3) and miR-33b (right, n = 3) in HepG2 cells transfected with the selected AMOs against miR-33a and miR-33b at a concentration of 5 nM. One-way ANOVA with Dunnett’s multiple comparison test. *P < 0.01. (**f**) Absolute copy number of miR-33a and miR-33b in THP-1 macrophages with or without tumor necrosis factor α (TNF-α) treatment. Two-way ANOVA with Holm–Sidak’s multiple comparisons test. *P < 0.05, **P < 0.01 and ***P < 0.001. (**g**) Expression levels of *sterol regulatory element-binding transcription factor* (*SREBF1*) and ATP binding cassette transporter A1 (*ABCA1*) in THP-1 macrophages with or without TNF-α treatment determined using quantitative real-time PCR. Two-way ANOVA with Holm–Sidak’s multiple comparisons test. **P < 0.01 and ***P < 0.001. (**h**) Absolute copy number of miR-33a and miR-33b in HASMCs and EA.hy926 with or without TNF-α treatment (right). Expression levels of *SREBF1* and *ABCA1* in HASMCs and EA.hy926 with or without TNF-α treatment (left) determined using quantitative real-time PCR. Two-way ANOVA with Holm–Sidak’s multiple comparisons test. *P < 0.05, **P < 0.01 and ***P < 0.001. ns is defined as not significant. All data represent mean ± SEM. Incubation with TNF-α was as follows: 3 h at 25 ng/mL for mouse peritoneal macrophages (PMs), 4 h at 10 ng/mL for mouse vascular smooth muscle cells (VSMCs), and 1 h at 50 ng/mL for mouse aortic endothelial cells (ECs).

We have generated luciferase reporter constructs (Fig. 2a) that had complementary sequences to miR-33a and miR-33b and designated them as miR-33a perfect match (miR-33a PM) and miR-33b PM, respectively. As a control, we utilized the empty vector that had neither sequence. As shown in Fig. 2b, reduction of luciferase activities in HepG2 cells transfected with miR-33a PM or miR-33b PM compared with the empty vector was achieved with the presence of endogenous miR-33a or miR-33b in HepG2 cells. Then, we tried to observe whether the luciferase activities changed in accordance with the different levels of intracellular miR-33a or miR-33b. For this purpose, we transduced different levels of miR-33a mimic or miR-33b mimic (33a mimic or 33b mimic) into HepG2 cells harboring either miR-33a PM or miR-33b PM. As indicated in Fig. 2c, luciferase activities were reduced when miR-33a PM or miR-33b PM were co-transfected with 33a mimic or 33b mimic, respectively. However, these changes in luciferase activity were small when miR-33a PM was expressed simultaneously with miR-33b mimic or when miR-33b PM was expressed simultaneously with miR-33a mimic. These results indicated that miR-33a PM and miR-33b PM specifically bind to miR-33a mimic and miR-33b mimic. Next, we measured the activities of the AMOs, anti-miR-33a and anti-miR-33b oligonucleotides using the increase in luciferase activity after transfection of these oligonucleotides into HepG2 cells harboring miR-33a PM or miR-33b PM. We generated 6 different anti-miR-33a oligonucleotides and 6 anti-miR-33b oligonucleotides, as shown in Supplementary Table S1 online. To increase the binding affinity of these to miR-33a and miR-33b, amido-bridged nucleic acids (AmNAs) were used for the synthesis of AMOs^17,18^. As a control, we generated a control AmNA containing a random sequence. The changes in luciferase activity in HepG2 cells transfected with 1 nM and 10 nM anti-miR-33a or anti-miR-33b oligonucleotides are indicated in Fig. 2d. The results showed that the AMOs 33a-1[16] and 33a-2[12] acted specifically on miR-33a, and 33b-1[16] and 33b-2[12] acted specifically on miR-33b. For ease of synthesis, we decided to use 33a-2[12] and 33b-2[12] in the following experiments. Next, we measured the levels of miR-33a and miR-33b in HepG2 cells transfected with control oligonucleotides, 33a-2[12] and 33b-2[12] at 5 nM. Transfection of 33a-2[12] and 33b-2[12] specifically reduced the levels of miR-33a and miR-33b, respectively (Fig. 2e). Thus, we designated 33a-2[12] as anti-miR-33a and 33b-2[12] as anti-miR-33b, hereafter.

### Anti-miR-33a increases the expression levels of *SREBF1* and miR-33b

First, we checked the effects of anti-miR-33a and anti-miR-33b in human cells. When we examined the effects of these AMOs in THP-1-derived macrophages, at steady state and upon TNF treatment, we found that the levels of both miR-33a and miR-33b were decreased with anti-miR-33b, whereas anti-miR-33a markedly increased miR-33b expression (Fig. 2f). We confirmed the expression of *SREBF1*, the host gene of miR-33b, and *ABCA1*, the target gene of miR-33a/b. Because *SREBF1* was upregulated by anti-miR-33a, we concluded that the upregulation of miR-33b by anti-miR-33a was mediated by the increased expression of *SREBF1*. This phenomenon did not occur with anti-miR-33b, and changes in the target gene *ABCA1* also reflected the amount of total miR-33 after AMO administration (Fig. 2g). The same study was also conducted in other cell types that make up the aorta, such as HASMC and EA.hy926. Similar to THP-1 macrophages, anti-miR-33a showed a marked increase in miR-33b expression, whereas miR-33a and miR-33b were decreased in anti-miR-33b in these cells (Fig. 2h). The results were similar in experiments using peritoneal macrophages, aortic vascular smooth muscle cells, and aortic endothelial cells in miR-33b KI mice (Supplementary Fig. S1 online).

### Anti-miR-33b ameliorated AAA formation in humanized miR-33b KI mice

**Figure 3:**
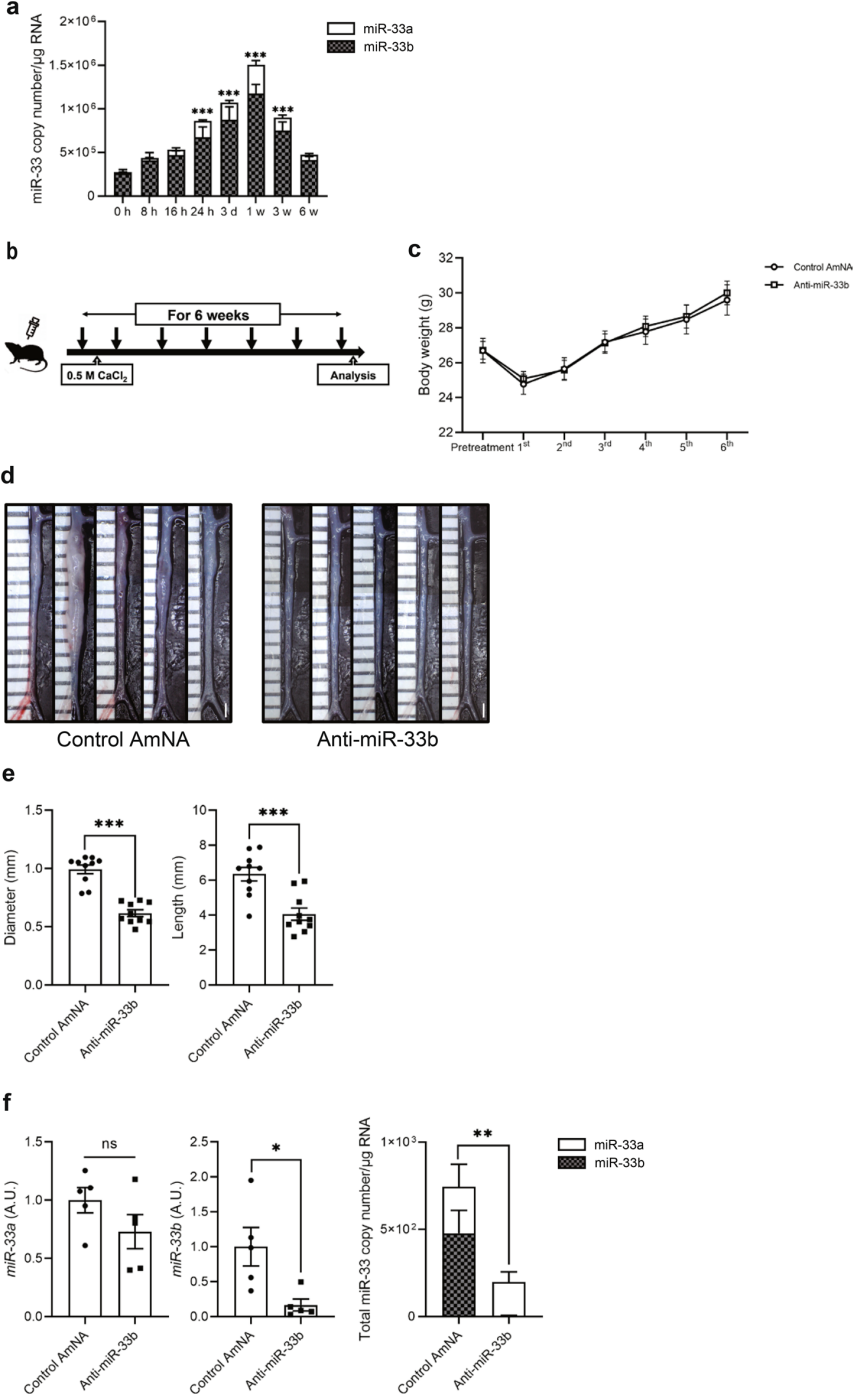
Beneficial effects of AMO administration against miR-33b to miR-33b KI mice for 6 weeks on CaCl_2_-induced AAA formation. (**a**) Copy number changes of miR-33a/b in abdominal aorta over a 6-week period, n = 8 mice in each group. One-way ANOVA with Dunnett’s multiple comparison test. ***P < 0.001 compared with baseline analysis. (**b**) Protocol scheme of administration of AMOs for 6 weeks. (**c**) Serial body weight changes during the experimental period, n = 20 mice with control AmNA administration, n = 19 mice with anti-miR-33a administration, and n = 28 mice with anti-miR-33b administration. (**d**) Representative photographs of CaCl_2_-induced AAA with administration of the indicated AMOs to miR-33b KI mice once a week for 6 weeks. White bars indicate 1 mm. (**e**) Maximum diameter and lesion length of abdominal aorta between left renal artery and terminal aorta of CaCl_2_-induced AAA, n = 10 mice in each group. Mann–Whitney test (left) and unpaired two-tailed t test (right). ***P < 0.001. (**f**) Expression levels of miR-33a (left) and miR-33b (mid), and absolute copy numbers of miR-33a and miR-33b (right) in the CaCl_2_-induced AAA of miR-33b KI mice with administration of the indicated AMOs, n = 5 mice in each group. Unpaired two-tailed t test (left and right) and Mann–Whitney test (mid). *P < 0.05 and **P < 0.01. ns is defined as not significant. All data represent mean ± SEM.

We examined the effects of anti-miR-33b in a CaCl_2_-induced mice model of AAA using humanized miR-33b KI mice. In this model, the copy numbers of both miR-33a/b increased, with a peak at 1 week, but most of the increase was in miR-33b (Fig. 3a). Copy numbers of miR-33a and miR-33b were also measured in human aortic aneurysm samples, and similarly, miR-33b copy numbers were increased especially in the center of the aortic aneurysm (Supplementary Fig. S2 online). We subcutaneously injected anti-miR-33b, or control oligonucleotides containing AmNA (Control AmNA, as shown in Supplementary Table S1) at a concentration of 10 mg/kg/week just before and after treatment with CaCl_2_ for 6 weeks (Fig. 3b). There was no difference in body weight among the 2 groups (Fig. 3c). As shown in Fig. 3d,e, treatment with anti-miR-33b significantly reduced the diameter and lesion length of the aorta compared with the control AmNA. When we measured the relative levels of miR-33a and miR-33b among these samples, miR-33a showed a downward trend, whereas miR-33b expression was significantly decreased in mice treated with anti-miR-33b (Fig. 3f). Notably, total miR-33 copy number (copy number of miR-33a and miR-33b) per 1 µg of total RNA was significantly reduced in mice treated with anti-miR-33b compared with the control group (Fig. 3f).

### Anti-miR-33b ameliorated AAA formation even in the acute phase of AAA formation in miR-33b KI mice

**Figure 4:**
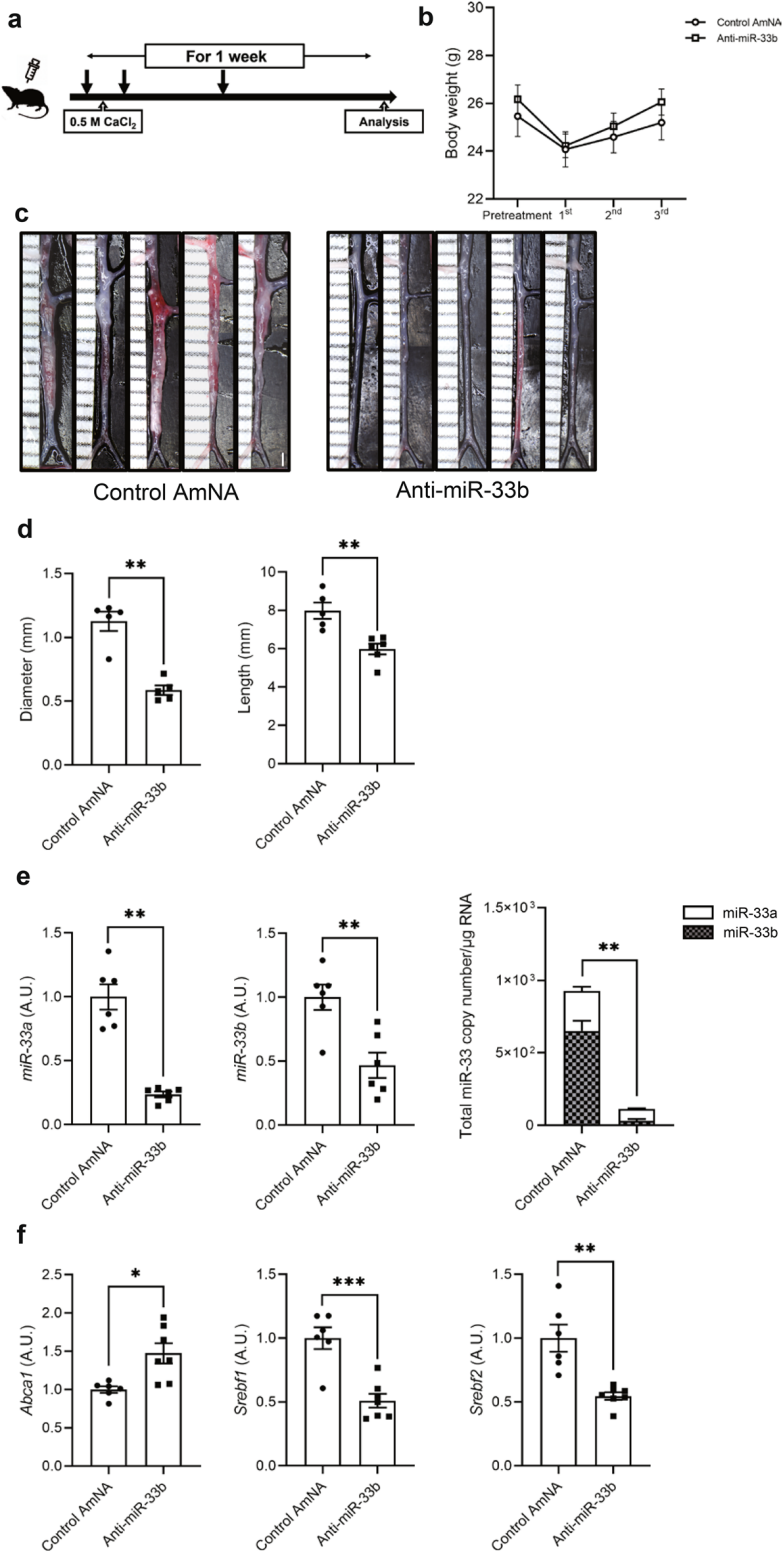

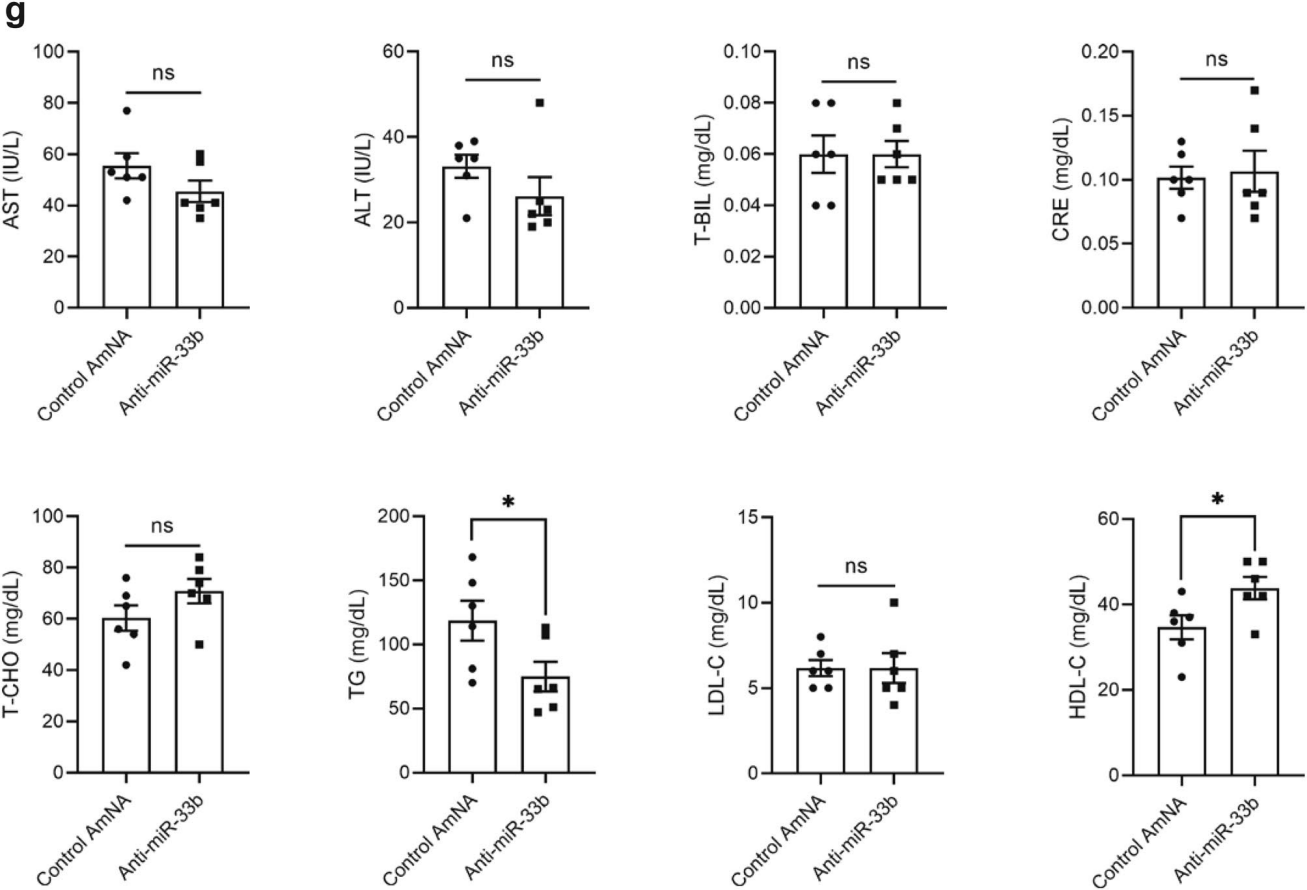
Acute beneficial effects of AMO administration against miR-33b to miR-33b KI mice for 1 week on CaCl_2_-induced AAA formation. (**a**) Protocol scheme of the administration of AMOs for 1 week. (**b**) Serial body weight changes during the experimental period, n = 6 mice in each group. (**c**) Representative photographs of CaCl_2_-induced AAA with administration of the indicated AMOs to miR-33b KI mice for 1 week. White bars indicate 1 mm. (**d**) Maximum diameter and lesion length of the abdominal aorta between the left renal artery and terminal aorta of CaCl_2_-induced AAA, n = 5 mice in each group. Mann–Whitney test (left) and unpaired two-tailed t test (right). **P < 0.01. (**e**) Expression levels of miR-33a (left) and miR-33b (mid), and absolute copy numbers of miR-33a and miR-33b (right) in CaCl_2_-induced AAA of miR-33b KI mice with administration of the indicated AMOs, n = 6 mice in each group. Unpaired t test with Welch’s correction (left and right) and unpaired two-tailed t test (mid). **P < 0.01. (**f**) Expression levels of *Abca1* (left), *Srebf1* (mid), and *Srebf2* (right) in CaCl_2_-induced AAA of miR-33b KI mice with administration of the indicated AMOs, n = 6 mice in control amido-bridged nucleic acid (AmNA) administration and n = 7 mice in the anti-miR-33b administration. Unpaired t test with Welch’s correction (left and right) and unpaired two-tailed t test (mid). *P < 0.05, **P < 0.01 and ***P < 0.001. (**g**) Serum profiling of miR-33b KI mice with CaCl_2_-induced AAA formation and administration of the indicated ASOs for 1 week. Blood was obtained from 9-week-old male mice. Mann–Whitney test [comparison of aspartate transaminase (AST), alanine aminotransferase (ALT) and creatinine (CRE) value] and unpaired two-tailed t test [comparison of total bilirubin (T-BIL), total cholesterol (T-CHO), triglycerides (TG), low-density lipoprotein-cholesterol (LDL-C) and high-density lipoprotein cholesterol (HDL-C) value]. *P < 0.05 compared with control AmNA-administered group. ns is defined as not significant. All data represent mean ± SEM.

In a previous experiment, inflammatory reactions had subsided by 6 weeks after operation (data not shown). Therefore, we observed the effect of anti-miR-33b at 1 week after surgery. We subcutaneously injected anti-miR-33b at 10 mg/kg before, just after, and 3 days after operation, and then analyses were conducted at 1 week (Fig. 4a). There was no difference in body weight among the groups (Fig. 4b). As shown in Fig. 4c,d, the diameter and length of AAA were significantly smaller in the mice treated with anti-miR-33b compared with those treated with the control AmNA. These results were consistent with those analyzed at 6 weeks after operation. The relative amount of miR-33a and miR-33b was significantly suppressed by anti-miR-33b compared with the control AmNA at 1 week after surgery. Notably, total miR-33 copy number was significantly reduced in mice treated with anti-miR-33b, as observed in the analysis at 6 weeks after surgery (Fig. 4e). Furthermore, the level of *Abca1*, the main target of miR-33a and miR-33b, was increased in mice treated with anti-miR-33b, and the levels of *Srebf1* and *Srebf2* were consistent with the amounts of miR-33b and miR-33a in Fig. 4e,f. The serum profile of AMO-treated miR-33b KI AAA mice in the 1-week analysis is shown in Fig. 4g. Serum triglyceride level was reduced and serum HDL-C level was increased in mice treated with anti-miR-33b. These results indicated that anti-miR-33b acted protectively against AAA in the acute to subacute phase.

### Histological changes in the aorta after treatment with AMOs against miR-33b

**Figure 5:**
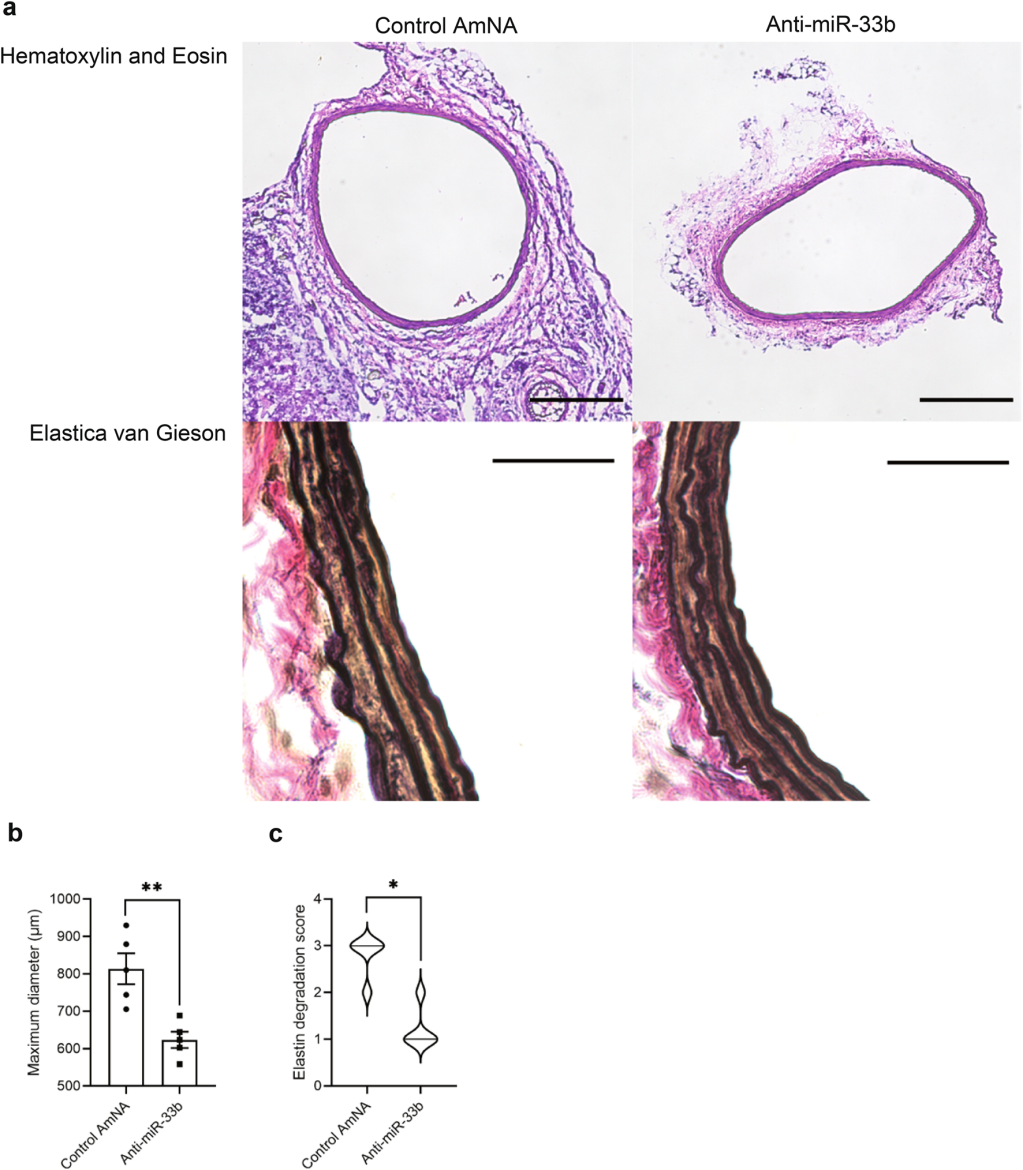
Histological analyses of AMO-administered CaCl_2_-induced AAA of miR-33b KI mice. (**a**) Representative images of hematoxylin–eosin (HE) and Elastica van Gieson (EVG) staining in CaCl_2_-induced AAA of miR-33b KI mice with administration of the indicated AMOs. Scale bar for HE, 200 µm; for EVG, 20 µm. (**b**) Maximum diameter of CaCl_2_-induced AAA with administration of the indicated AMOs, n = 5 in each group. Unpaired two-tailed t test. **P < 0.01. (**c**) The elastin degradation score of CaCl_2_-induced AAA with administration of the indicated AMOs, n = 5 in each group. Mann–Whitney test. *P < 0.05. All data represent mean ± SEM.

As shown in Fig. 5a, hematoxylin and eosin staining indicated a smaller aorta diameter in mice treated with anti-miR-33b. Elastica van Gieson staining showed fragmentation of the elastic layers of the aorta in samples treated with the control AmNA. A wavy form of the elastic layers remained in mice treated with anti-miR-33b. The maximum diameter of AAA and elastin degradation score^19^ were improved in mice treated with anti-miR-33b compared with those treated with the control AmNA (Fig. 5b,c).

### Macrophage accumulation and matrix metalloproteinase expression levels were lower in aortic samples treated with anti-miR-33b in miR-33b KI mice

**Figure 6:**
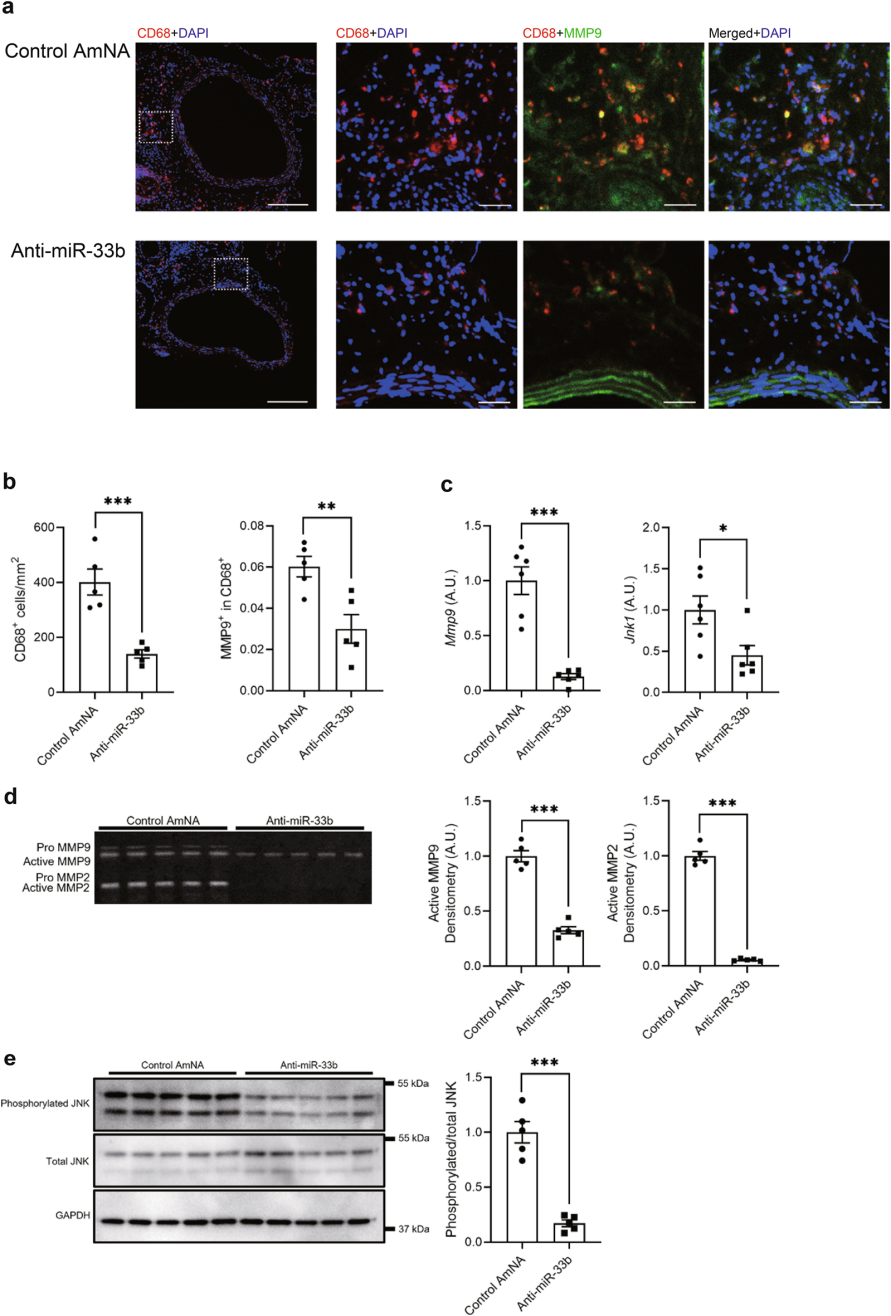
Inhibition of miR-33b in miR-33b KI mice prevents accumulation of macrophages and attenuates the activities of c-Jun N-terminal kinase (JNK) and matrix metalloproteinase 9 (MMP9). (**a**) Representative images of immunofluorescence staining for CD68 (red), MMP9 (green), and 4',6-diamidino-2-phenylindole (DAPI; blue). Scale bar indicates 200 µm for low power-field and 50 µm for high power-field. (**b**) Numbers of CD68-positive cells and quantification of CD-68/MMP9-double positive area in the sections of CaCl_2_-induced AAA wall, n = 5 in each group. Unpaired two-tailed t test. **P < 0.01 and ***P < 0.001. (**c**) *Mmp9* and *Jnk1* expression levels in CaCl_2_-induced AAA walls by quantitative real-time PCR, n = 6 mice in each group. Unpaired t test with Welch’s correction (left) and unpaired two-tailed t test (right). *P < 0.05 and ***P < 0.001. (**d**) MMP9 and MMP2 activities of AMO-administered CaCl_2_-induced AAA walls were evaluated using gelatin zymography (left) and quantified using densitometric analyses (right), n = 5 mice in each group. Original gel is presented in Supplementary Fig. S4 online. Unpaired two-tailed t test (active MMP9 densitometry analysis) and unpaired t test with Welch’s correction (MMP2 densitometry analysis). ***P < 0.001. (**e**) JNK activity of AMO-administered CaCl_2_-induced AAA walls was assessed using western blotting (left) and quantified using densitometric analyses (right). GAPDH was served as an internal loading control, n = 5 mice in each group. Original blots are presented in Supplementary Fig. S4 online. Unpaired t test with Welch’s correction. ***P < 0.001. All data represent mean ± SEM.

Next, we performed immunohistochemical analysis on the aortas in mice treated with anti-miR-33b or the control AmNA. As shown in Fig. 6a, although matrix metalloproteinase (MMP) 9- and CD68-positive cells accumulated in the aorta of mice treated with the control AmNA, such accumulation was suppressed in mice treated with anti-miR-33b. As shown in Fig. 6b, the number of CD68- and MMP9-positive cells was significantly lower in mice treated with anti-miR-33b than in mice treated with the control AmNA. Low magnification images of immunofluorescence staining for CD68, MMP9, and 4',6-diamidino-2-phenylindole (DAPI) are shown in Supplementary Fig. S3 online. Moreover, the mRNA expression levels of *Mmp9* and *Jnk1* showed similar patterns to the accumulation of CD68-positive cells (Fig. 6c). MMP9 and MMP2 activities in the aortas of mice treated with AMOs were examined using gelatin zymography, and MMP9 and MMP2 activities were significantly lower in mice treated with anti-miR-33b than in mice treated with the control AmNA (Fig. 6d and Supplementary Fig. S4 online). Phosphorylated c-Jun N-terminal kinases (JNKs)/total JNKs in aortas were significantly lower in mice treated with anti-miR-33b than in mice treated with control AmNA (Fig. 6e and Supplementary Fig. S4 online).

### Monocyte chemotactic protein-1 and other cytokines expressions were lower in aortic samples treated with anti-miR-33b in miR-33b KI mice

**Figure 7:**
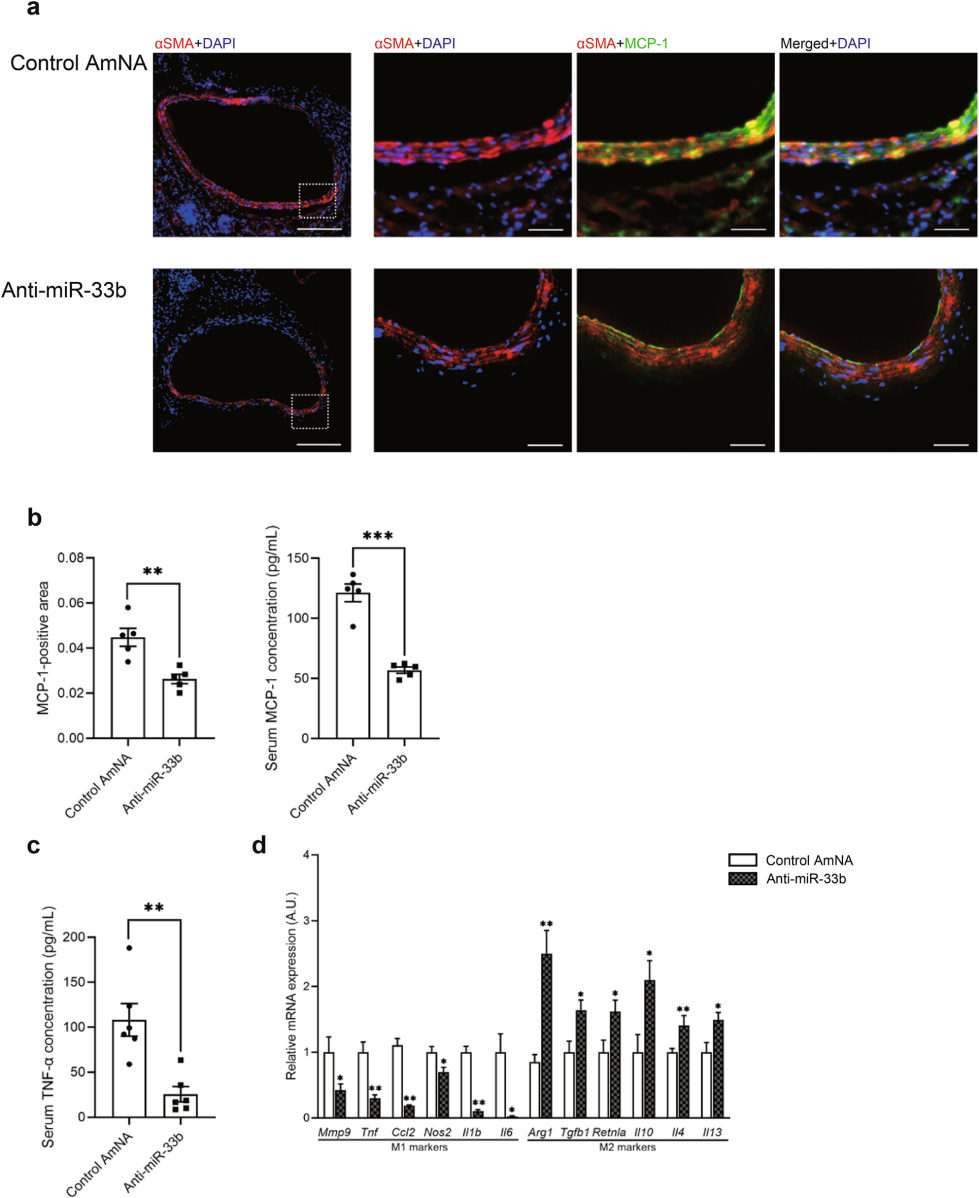
Inhibition of miR-33b in miR-33b KI mice prevents the secretion of MCP-1 and conserves the structure of VSMCs. (**a**) Representative images of immunofluorescence staining for αSMA (red), MCP-1 (green), and DAPI (blue). Scale bar indicates 200 µm for low power-field and 50 µm for high power-field. (**b**) Quantification of the MCP-1-positive area in sections of the CaCl_2_-induced AAA wall (left) and serum MCP-1 concentration using an enzyme-linked immunosorbent assay (right), n = 5 mice for immunofluorescence staining and enzyme-linked immunosorbent assay. Unpaired two-tailed t test (left and right). **P < 0.01 and ***P < 0.001. (**c**) Serum TNF-α concentration of CaCl_2_-induced AAA mice with administration of the indicated AMOs, n = 6 mice in each group. Unpaired two-tailed t test. **P < 0.01. (**d**) Real-time PCR analysis of classical M1 and M2 markers of CaCl_2_-induced AAA mice with administration of the indicated AMOs, n = 6 mice in each group. Unpaired two-tailed t test (*Mmp9*, *Tnf*, *Ccl2*, *Nos2*, *Arg1*, *Tgfb1*, *Retnla*, *Il10* and *Il13*) and Mann–Whitney test (*Il1b*, *Il6* and *Il4*). *P < 0.05 and **P < 0.01. All data represent mean ± SEM.

As indicated in Fig. 7a, monocyte chemotactic protein-1 (MCP-1) was preferentially expressed in α-smooth muscle actin (αSMA)-positive cells in mice treated with control AmNA. MCP-1-positive areas and serum MCP-1 levels were significantly lower in mice treated with anti-miR-33b than in control AmNA-treated mice (Fig. 7b). Furthermore, serum tumor necrosis factor α (TNF-α) levels were significantly lower in mice treated with anti-miR-33b than in mice treated with control AmNA (Fig. 7c). Low magnification images of immunofluorescence staining of MCP-1, αSMA, and DAPI are shown in Supplementary Fig. S5 online. It was previously reported that inhibition of miR-33 altered macrophage metabolism, resulting in a shift of macrophage polarity to M2 and exerting anti-inflammatory effects^20^. Therefore, we have examined the polarization of macrophages using the anti-miR-33b-treated aorta samples. The results showed that anti-miR-33b induced a shift of macrophages to M2 (Fig. 7d), similar to a previous paper^20^.

### Anti-inflammatory effects were observed in CaCl_2_-induced AAA samples in anti-miR-33b-treated miR-33b KI mice

**Figure 8:**
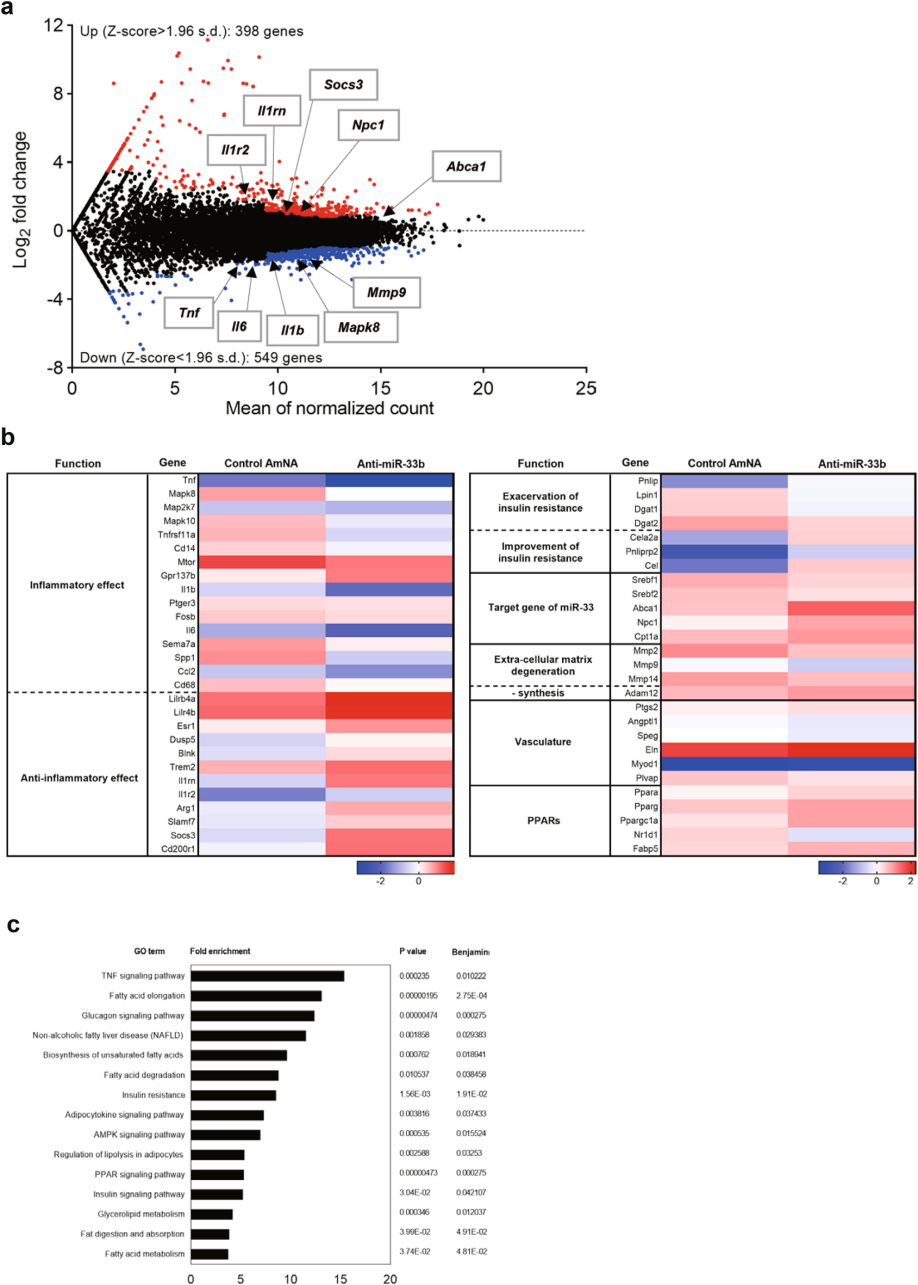

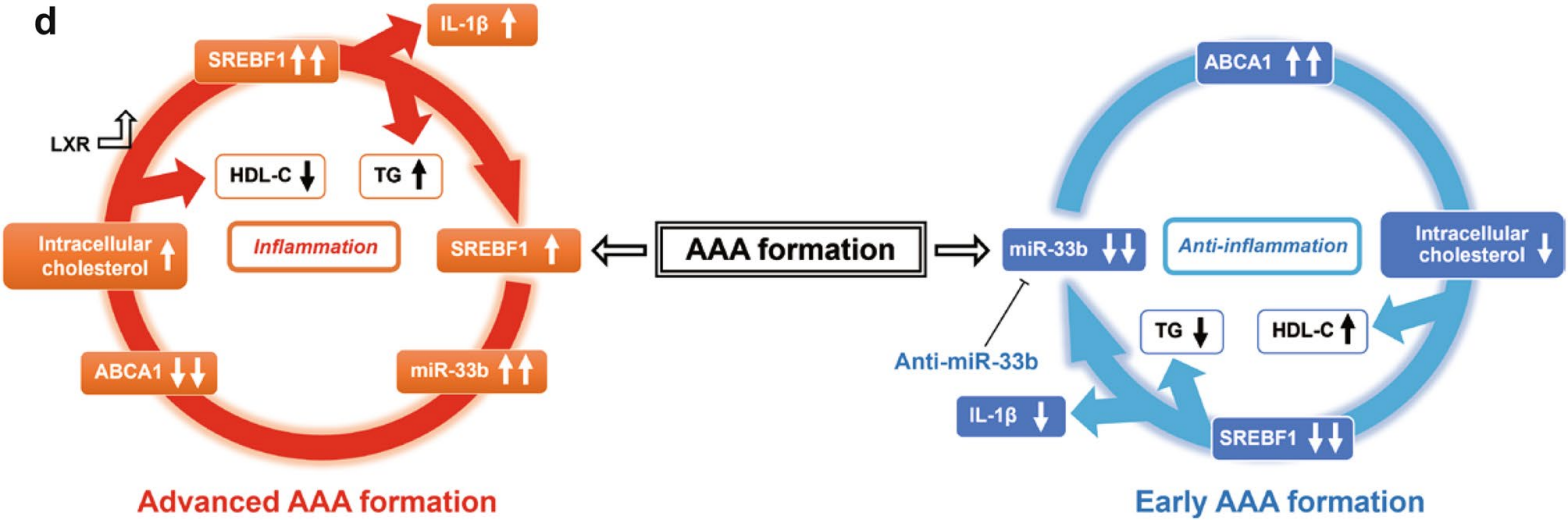
RNA-seq analysis for CaCl_2_-induced AAA samples of AMO-administered miR-33b KI mice and anti-miR-33b working hypothesis. (**a**) MA plot illustrating the number of differentially expressed genes. Red dots: upregulated genes; blue dots: downregulated genes, as determined by log_2_(fold change) between anti-miR-33b and control AmNA administered CaCl_2_-induced AAA samples of miR-33b KI mice. (**b**) Heatmap representation of normalized gene expressions as intensity-based Z-score. (**c**) Enrichment analyses of integrated pathways for anti-miR-33b injected AAA miR-33b KI mice. (**d**) Schematic diagram of the effects of anti-miR-33b.

RNA-seq analysis was performed on the aortas of miR-33b KI AAA mice treated with AMOs. First, the data were presented on the relationship between the original gene expression levels and the increase or decrease in gene expression with anti-miR-33b (Fig. 8a). Next, the data were presented on the increase or decrease in gene expression for each gene function. The expression of a group of genes that trigger inflammatory responses was suppressed by anti-miR-33b. On the other hand, the expression levels of genes with anti-inflammatory effects, such as interleukin (IL)-1 receptor antagonist (IL-1rn), were upregulated in anti-miR-33b-treated miR-33b KI AAA mice (Fig. 8b). In pathway enrichment analysis, changes in inflammation and lipid pathways were observed: the gene clusters comprising the TNF signaling pathway were downregulated in anti-miR-33b-treated miR-33b KI AAA mice (Fig. 8c). Moreover, the target genes of miR-33 whose gene expression was increased by anti-miR-33b were identified by RNA-seq, and the results were further confirmed by qPCR (Supplementary Fig. S6a,b online). As expected, *Prkaa1*, *Cpt1a*, *Crot*, and *Hadhb* were upregulated in anti-miR-33b-treated aorta samples^21,22^. All of these are the direct targets of miR-33 and it is likely that these changes are enhancing fatty acid oxidation, leading to M2 macrophage polarization and a reduced inflammatory response^20^. Our experimental results indicated that severe inflammation is induced in AAA formation. In this situation, the expression of *Srebf1* and miR-33b was increased, and the expression of *Abca1*, a target gene of miR-33b, was decreased. Therefore, intracellular cholesterol increases resulted in a further increase in *Srebf1* via LXR transcriptional activation. Furthermore, it is known that *Srebf1* promotes the production of IL-1^23,24^. These changes are thought to form a vicious cycle that leads to advanced AAA formation. When anti-miR-33b was administered, the amount of miR-33b was suppressed, and thus the vicious cycle caused by miR-33b does not occur. Furthermore, the total amount of miR-33 was reduced and the *Abca1* level was increased, thereby suppressing aortic inflammation. At the same time, the amount of intracellular cholesterol was reduced and *Srebf1* further suppressed. Improved lipid profile such as HDL-C elevation and TG reduction can both have an additional anti-inflammatory effect. These findings suggested that anti-miR-33b treatment results in the suppression of AAA (Fig. 8d).

## Discussion

In this study, we obtained the following results. 1. In experiments using genetically modified mice, WT mice with only miR-33a, KOKI mice with only miR-33b^15^, and mice with both miR-33a and miR-33b^14^ were subjected to aneurysm formation using the CaCl_2_ method^16^, and the mice deteriorated progressively in proportion to the total amount of miR-33. 2. We created 12-base AMOs (anti-miR-33a and anti-miR-33b) specific for miR-33a and miR-33b, respectively, which were able to inhibit miR-33a and miR-33b individually in in vitro experiments. 3. Anti-miR-33b treatment markedly improved aortic aneurysm induced using the CaCl_2_ method compared with a control AMO.

In the CaCl_2_-induced AAA model of genetically engineered mice, AAA formation was worse in mice expressing only miR-33b (KOKI) than in wild-type mice with only miR-33a (WT). Furthermore, KI mice expressing both miR-33a and miR-33b showed more severe AAA formation than mice expressing only miR-33b (KOKI). The total miR-33 copy number increased in the order WT, KOKI, and KI, and the majority of the increase was in miR-33b. This suggested that miR-33b has a significant effect on aortic aneurysm formation.

For in vitro experiments, human cell lines that were thought to be major cell types of AAA indicated that the levels of *SREBF1* and total miR-33 copy numbers increased and the levels of *ABCA1* decreased after treatment with anti-miR-33a. On the contrary, the expression levels of *SREBF1* and total miR-33 copy numbers decreased and the expression levels of *ABCA1* increased with anti-miR-33b treatment in these human cell lines (Fig. 2f,g). Similar results were shown in peritoneal macrophages, aortic vascular muscle cells, and aortic endothelial cells in miR-33b KI mice (Supplementary Fig. S1a,b online). However, the exact mechanism of these responses in cells treated with anti-miR-33a is currently unknown and needs to be elucidated in the future. It is known that ABCA1 has anti-inflammatory effects^25,26^ and *SREBF1* has potency for the induction of IL-1β^23,24^. Based on the above, we planned to conduct comparison experiments between anti-miR-33b and control AmNA to investigate the effect of anti-miR-33b on a CaCl_2_-induced AAA murine model in detail.

In the model that we used, both miR-33a and miR-33b increased during the 6 weeks of aortic aneurysm formation, with a peak at 1 week, but miR-33b accounted for the majority of the increase when copy number was measured (Fig. 3a), which may be one reason why the effect of anti-miR-33b was so pronounced. In mice treated with anti-miR-33b, there was an increase in serum HDL-C and decreases in serum triglyceride, macrophage accumulation and MMP9 expression, phosphorylated JNK, MCP-1 expression in smooth muscle cells, and in pro-inflammatory cytokine expression in the aorta (Figs. 4, 5, 6 and 7), which was similar to a previous study using genetically modified mice^7^.

In AAA formation, especially in inflammation-stimulated macrophages, SREBP-1 is activated via processing from the endoplasmic reticulum membrane during the inflammatory response between12 and 24 h after stimulation^27^. For this reason, the role of SREBP-1 and miR-33b may be even greater in acute inflammation. miR-33b increases intracellular cholesterol levels by decreasing levels of ABCA1, which may further stimulate levels of *Srebf1* and miR-33b via activation of LXRs (a vicious circle caused by miR-33b). *Srebf1* is known to promote IL-1β production^23,24^ and may have exacerbated inflammation in AAA formation. On the other hand, in the case of anti-miR-33b treatment, induction of miR-33b was suppressed, suggesting that the vicious cycle caused by miR-33b did not occur in the first place. Furthermore, the decrease in the total amount of miR-33 and the increase in ABCA1 levels may have suppressed inflammation in the aorta. At the same time, intracellular cholesterol levels may have been reduced, which may have further suppressed *Srebf1*. In addition, an improved serum lipid profile was observed with anti-miR-33b treatment. Elevated HDL-C and reduced TG levels, which may be medicated by increased ABCA1 and decreased SREBF1 respectively, are likely to have an additional anti-inflammatory effect^28–33^. Therefore, suppression of miR-33b may be effective in the treatment of aortic aneurysms, for which there is currently no clear treatment (Fig. 8c). This hypothesis may be similar to that of mice expressing only miR-33a (WT mice), which showed less AAA formation than mice expressing only miR-33b (KOKI mice).

Furthermore, RNA-seq analysis of AMOs-injected KI AAA mice also showed that gene expression in the TNF signaling pathway was decreased in miR-33b KI AAA mice injected with anti-miR-33b (Fig. 8a–c). miR-33b KI AAA mice injected with anti-miR-33b showed increased expression of genes with anti-inflammatory effects, such as IL-1rn, which supports the above idea. To further clarify why the inflammatory pathway is suppressed by anti-miR-33b in this experiment, we have also examined the polarization of macrophages using post-treatment aorta samples. The results showed that anti-miR-33b induced a shift in macrophages to M2 (Fig. 7d), similar to a previous paper^20^. Moreover, *Prkaa1*, *Cpt1a*, *Crot*, and *Hadhb* expression levels were increased by treatment with anti-miR-33b (Supplementary Fig. S6b online), which may indicate this polarization of macrophages to the M2 phenotype is caused by increased fatty acid oxidation^21,22^. Thus, it is possible that changes in macrophage metabolism may work to suppress macrophage inflammation.

In the present study, AmNA was used to generate AMOs because this nucleic acid is known to greatly reduce hepatotoxicity compared with conventional locked nucleic acids^17,18^. No obvious liver and renal injury was observed in the present study. In our study, the administration of AMOs was started just before the creation of the model to confirm the maximum effect. Therefore, anti-miR-33b may have been able to exert its effect by suppressing inflammation in the acute phase. In clinical cases, treatment of aneurysms that have already progressed to a certain degree is considered to be the main focus. Therefore, it is desirable to be able to confirm the effects in the chronic phase and over a long-term period in an animal model in which the aneurysm diameter expands gradually. Currently, several experimental mouse models of AAA have been reported^34–37^. Confirmation of the anti-miR-33b AMO effect using another AAA model may be useful for further understanding of the mechanisms. On the other hand, there are currently nucleic acid drugs for dyslipidemia that have shown efficacy when administered once every 6 months^38–40^. If the anti-miR-33b used in the present study was also found to have long-term effects, it may be developed as a drug that is closer to clinical application.

The development of a new treatment for aortic aneurysms, for which no clear treatment has been established at present, is an urgent issue. This study suggests that suppression of miR-33b may be effective in the treatment of AAA, perhaps by a mechanism that suppresses inflammation through changes in intracellular metabolism. The efficacy of anti-miR-33b in clinical cases should be investigated in the future.

## Methods

### Animals

miR-33b knock-in (KI) mice were generated as described previously^14^. miR-33a knockout miR-33b knock-in mice (KOKI) were described previously^15^. WT littermates were used as controls. Every strain used had the C57BL/6J background. For the CaCl_2_-induced AAA model, periaortic application of CaCl_2_ was performed as described previously^16^. Briefly, 8-week-old male mice were anesthetized (intraperitoneal injection of three types of mixed anesthetic agents: medetomidine 0.3 mg/kg body weight at a concentration of 1.0 mg/mL; midazolam 4.0 mg/kg body weight at a concentration of 5.0 mg/mL; and butorphanol 1.0 mg/kg body weight at a concentration of 5.0 mg/mL) and underwent laparotomy. The abdominal aorta between the renal arteries and the bifurcation of the iliac arteries was isolated from the surrounding retroperitoneal structures. Then, 0.5 mol/L CaCl_2_ was applied to the external surface of the aorta. Saline was substituted for CaCl_2_ in sham control mice. After 20 min, the abdominal aorta was rinsed with saline and the incision was closed. AAA diameter was assessed 1 week or 6 weeks after the procedure.

All mice were maintained in specific-pathogen-free laboratories at Kyoto University Graduate School of Medicine. This investigation was performed with the approval of the Kyoto University Ethics Review Board. All mice received humane care in accordance with the ARRIVE (Animal Research: Reporting of In Vivo Experiments) guidelines developed by the National Centre for the Replacement, Refinement, and Reduction of Animals in Research. All methods were carried out in accordance with relevant guidelines and regulations. Every effort was made to minimize animal suffering and to reduce the number of mice used.

### Human AAA samples

This investigation was performed with the approval of the Kyoto University Ethics Review Board. Tissue samples were collected from patients who underwent open surgery for AAA between 2017 and 2018. Tissue samples were taken from the anterior wall of the aneurysm body in a cord-like fashion from the normal border region to the largest enormous area.

All protocols conformed to the ethical guidelines of the Declaration of Helsinki and samples were obtained after receiving written informed consent.

### Cell culture and reagents

THP-1 cells (TIB-202™, ATCC) were cultured in RPMI1640 (Nacalai Tesque) containing 10% fetal bovine serum (FBS). THP-1 macrophages were transformed into macrophages by incubation for 3 days with 100 nM PMA (Nacalai Tesque). HASMC cells (purchased from KURABO Bio-Medical, KS-4009) were cultured in HuMedia-SG2 (KS-2170S, KURABO Bio-Medical). EA.hy926 cells (CRL-2922™, ATCC) were cultured in Dulbecco’s modified Eagle’s medium (DMEM; Nacalai Tesque, Japan) supplemented with 10% FBS.

miR-33b KI mouse peritoneal macrophages were obtained from the peritoneal cavity of mice 4 days after intraperitoneal injection of 3 mL of 3% thioglycollate. The cells obtained were washed, spun at 1000 rpm for 5 min, and plated in RPMI1640 containing 10% FBS. miR-33b KI mouse smooth muscle cells and aortic endothelial cells were isolated using the established method^41^. Briefly, the aorta of mice was surgically isolated and rinsed with phosphate-buffered saline (PBS) containing 1000 U/mL heparin (FUJIFILM Wako Special Grade) and immersed in 20% FBS-DMEM containing 1000 U/mL heparin. The connecting tissue was quickly removed with the use of a stereoscopic microscope. The inside of the aortic lumen was washed with serum-free DMEM, filled with 2 mg/mL type II Collagenase (CLS2, Worthington), and incubated at 37 °C for 45 min. Endothelial cells were removed by flushing with 5 mL 20% FBS-DMEM and collected by centrifuging at 1200 rpm for 5 min. The cells were then suspended gently with 20% FBS-DMEM and seeded in type I collagen-coated 35 mm dishes. The cleaned aorta was cut into 2 mm rings and immediately placed in 10% FBS-DMEM. The samples were immediately transferred to serum-free DMEM with 2 mg/mL type II Collagenase and incubated at 37 °C for 2 h. Smooth muscle cells were collected by centrifuging at 1200 rpm for 5 min and maintained in 10% FBS-DMEM.

For AMO transfection into THP-1 macrophages, HASMC, EA.hy926, mouse peritoneal macrophages, mouse vascular smooth muscle cells, and mouse aortic endothelial cells, Lipofectamine™ RNAiMAX Transfection Reagent (Thermo Fisher Scientific, Japan) was used in accordance with the manufacturer’s instructions.

### Screening for AMOs against miR-33a and miR-33b

As shown in Supplementary Table S1 online, we designed 6 different anti-miR-33a oligonucleotides and 6 anti-miR-33b oligonucleotides containing AmNA^17,18^. These anti-miR-33a or anti-miR-33b oligonucleotides were purchased from GeneDesign (Osaka, Japan). To assess the inhibition efficacy and specificity, we constructed two reporter vectors harboring miR-33a/miR-33b perfect match sequences using psiCHECK2-let-7 8 × (https://www.addgene.org/20931). We transfected these constructs with AMOs into HepG2 (HB-8065™, ATCC) cells at the indicated concentrations using Lipofectamine 2000. After incubation for 24 h, the luciferase activities were measured using a PicaGeneR Dual SeaPansy™ Luminescence Kit (TOYOBO).

### Administration of AMOs against miR-33b and control AmNA

Eight-week-old male miR-33b KI mice were utilized for examinations. For analysis after 6 weeks, AMOs at 10 mg/kg body weight were administered the day before and the next day after operation, and once a week for 6 weeks from that point. These mice were analyzed 1 week after the last administration. For analysis after 1 week, AMOs were administered on the day before and the next day after, and 3 days after operation. AMOs were injected subcutaneously into the posterior region of the neck.

### Serum biochemical analysis

Blood was obtained from the inferior vena cava of anesthetized mice, and serum was separated by centrifugation at 4 °C and stored at -80 °C. Employing standard methods, biochemical measurements were made using a Hitachi 7180 Auto Analyzer (Nagahama Life Science Laboratory, Nagahama, Japan).

### RNA extraction and quantitative real-time PCR

Total RNA was isolated and purified using Tripure Isolation Reagent (Roche), and cDNA was synthesized from 1 µg of total RNA using a Verso cDNA Synthesis Kit (Thermo Fisher) in accordance with the manufacturer’s instructions. For quantitative RT-PCR, specific genes were amplified using 40 cycles with THUNDERBIRD SYBR qPCR Mix (TOYOBO). Products were analyzed using StepOnePlus (Applied Biosystems). Expression was normalized to the housekeeping gene β-actin or 18S. Gene-specific primers are listed in Supplementary Table S2 online.

### Quantitative PCR for microRNAs

miR-33a and miR-33b were measured using TaqMan MicroRNA assay protocols (Applied Biosystems). Products were analyzed using StepOnePlus (Applied Biosystems). miR expression in samples were normalized using U6 snRNA expression.

### Protein extraction and western blotting

Mice were perfused with cold PBS at physiological pressure, and aortic specimens were dissected using microscissors. Western blotting was performed using standard procedures as described previously^42^. Lysis buffer containing of 100 mmol/L Tris–HCl, 75 mmol/L NaCl, and 1% Triton X-100 (Nacalai Tesque) at pH 7.4 was supplemented with Complete mini protease inhibitor (Roche), ALLN (25 µg/mL), 0.5 mM NaF, and 10 mM Na_3_VO_4_ just prior to use. Protein concentrations were determined using a bicinchoninic acid protein assay kit (Bio-Rad). All samples (20 µg of protein) were suspended in lysis buffer, fractionated using NuPAGE 4–12% Bis–Tris (Invitrogen) gels, and transferred to a Protran nitrocellulose transfer membrane (Whatman). The membrane was blocked using 1 × PBS containing 5% non-fat milk for 1 h and incubated with a primary antibody [anti-phospho-SAPK/JNK (1:1000; #9251, Cell Signaling), anti-SAPK/JNK (1:1000; #9252, Cell Signaling), anti-GAPDH (1:3000; #2118S, Cell Signaling)] overnight at 4 °C. After washing with PBS-0.05% Tween 20 (0.05% T-PBS), the membrane was incubated with the secondary antibody (anti-rabbit IgG horseradish peroxidase-linked; 1:2000) for 1 h at 4 °C. The membrane was then washed with 0.05% T-PBS and detected with an ECL Western Blotting Detection Reagent (GE Healthcare) using an Amersham Imager 680 (GE Healthcare).

### Gelatin zymography

Gelatin zymography was performed using a Gelatin-Zymography Kit (PMC-AK45-COS; Cosmo Bio, Japan) in accordance with to the manufacturer’s directions. The results were evaluated using densitometric analysis of the lytic areas obtained from the gelatin electrophoresis using ImageJ software (ver. 1.52a; National Institutes of Health).

### Tumor necrosis factor-α and monocyte chemotactic protein-1 enzyme-linked immunosorbent assay

Analysis was performed with 50 µL of mice serum concentrations of TNF-α and MCP-1 determined using a Quantikine ELISA kit (MTA00B; R&D Systems) in accordance with the manufacturer’s instructions.

### Immunohistochemistry

Mice were sacrificed using intraperitoneal injection of a sufficient amount of the mixed anesthetic agents and perfused with PBS and subsequently with 4% paraformaldehyde (WAKO, Japan) at physiological pressure. Then, aortic specimens were dissected, rinsed with cold PBS and immediately frozen in OCT compound on a block of dry ice. Sections (10 mm) were cut from the specimens. The frozen sections were washed three times with 0.05% PBS-T then covered with anti-CD68 antibody (1:200; MCA 1957, Serotec), anti-MMP9 antibody (1:50; AF909, R&D), anti-MCP-1 antibody (1:50; ab25124, abcam) or anti-αSMA antibody (1:200; 1A4, Sigma-Aldrich), and incubated at 4 °C overnight. The sections were washed three times with PBS-T and incubated with anti-rat secondary antibody for 30 min at room temperature. Then, the sections were rinsed again three times with PBS-T and covered with mounting medium with DAPI (H-1200, Vectashield). The positively stained areas of each aorta were measured using a digital fluorescence microscope (ZEISS ZEN Imaging Software). The number of immunoreactive cells was calculated using 5 different fields at magnification of × 50.

### RNA sequencing analysis

For RNA-seq, total RNA was isolated using Tripure Isolation Reagent (Roche) and purified using NucleoSpin RNA Plus (#740984.50, Macherey–Nagel). Sample preparation, normalization and sequence analysis were requested to DNA Chip Research Inc. (Tokyo, Japan).

### Statistics

Results are given as mean ± standard error of the mean (SEM). Distributions of all variables were tested using the Kolmogorov–Smirnov test. Equal variance between groups was tested using the F test. If the data were nonnormally distributed, t test with Welch’s correction was used. Mann–Whitney test was used in nonparametric data. One-way analysis of variance (ANOVA) and two-way ANOVA followed by Holm–Sidak’s multiple comparison test for the pairwise comparison test and Dunnett’s multiple comparison test for the versus control test were used for one-factorial experiments. A probability value of < 0.05 was considered statistically significant. Statistical analyses were conducted using Prism 8 (GraphPad Software, Inc. USA).

## Supplementary Information

Supplementary Information.

## Data Availability

The data underlying this article will be shared on reasonable request to the corresponding author. The datasets generated and analyzed during the current study are available in the DDBJ Sequence Read Archive repository (https://trace.ddbj.nig.ac.jp/) under the Accession number DRA013651 (https://trace.ddbj.nig.ac.jp/D-way/contents/dra/submission_detail?serial=1).

## Abbreviations

AAA: Abdominal aortic aneurysm
ABCA1: ATP binding cassette transporter A1
ALT: Alanine aminotransferase
AMO: Anti-microRNA oligonucleotide
AmNA: Amido-bridged nucleic acid
ANOVA: Analysis of variance
AST: Aspartate transaminase
CaCl_2_: Calcium chloride
CRE: Creatinine
DAPI: 4',6-Diamidino-2-phenylindole
DMEM: Dulbecco’s modified Eagle’s medium
EC: Endothelial cell
EVG: Elastica van Gieson
FBS: Fetal bovine serum
HDL-C: High-density lipoprotein cholesterol
HE: Hematoxylin–eosin
IL: Interleukin
JNK: C-Jun N-terminal kinase
KI: Knock-in
KO: Knock-out
KOKI: Knock-out, knock-in
LDL-C: Low-density lipoprotein-cholesterol
MCP-1: Monocyte chemotactic protein-1
miR: MicroRNA
MMP: Matrix metalloproteinase
PBS: Phosphate-buffered saline
PM: Peritoneal macrophage
SEM: Standard error of the mean
αSMA: α-smooth muscle actin
SREBF: Sterol regulatory element-binding transcription factor
T-BIL: Total bilirubin
T-CHO: Total cholesterol
TG: Triglycerides
TNFα: Tumor necrosis factor α
VSMC: Vascular smooth muscle cell
WT: Wild-type
