# Supplementary material for: Inhibition of microRNA-33b specifically ameliorates abdominal aortic aneurysm formation via suppression of inflammatory pathways
Source: Sci Rep. 2022 Jul 14;12:11984. doi: 10.1038/s41598-022-16017-5 (PMC9283493; doi:10.1038/s41598-022-16017-5)
Supplement: Supplementary file 1 — Supplementary Information. [file 41598_2022_16017_MOESM1_ESM.pdf]

Supplementary information

Inhibition of MicroRNA-33b Specifically Ameliorates Abdominal Aortic Aneurysm Formation via Suppression of Inflammatory Pathways

Tomohiro Yamasaki, MD; Takahiro Horie, MD, PhD; Satoshi Koyama, MD, PhD; Tetsushi Nakao, MD, PhD; Osamu Baba, MD, PhD; Masahiro Kimura, MD, PhD; Naoya Sowa, MS; Kazuhisa Sakamoto, MD; Kazuhiro Yamazaki, MD, PhD; Satoshi Obika, PhD; Yuuya Kasahara, PhD; Jun Kotera, PhD; Kozo Oka, PhD; Ryo Fujita, PhD; Takashi Sasaki, PhD; Akihiro Takemiya, PhD; Koji Hasegawa, MD, PhD; Kenji Minatoya, MD, PhD; Takeshi Kimura, MD, PhD; Koh Ono, MD, PhD

Supplementary Figure S1. Comparison of miR-33 target genes and total amount of miR-33 in miR-33b KI mice primary cells.

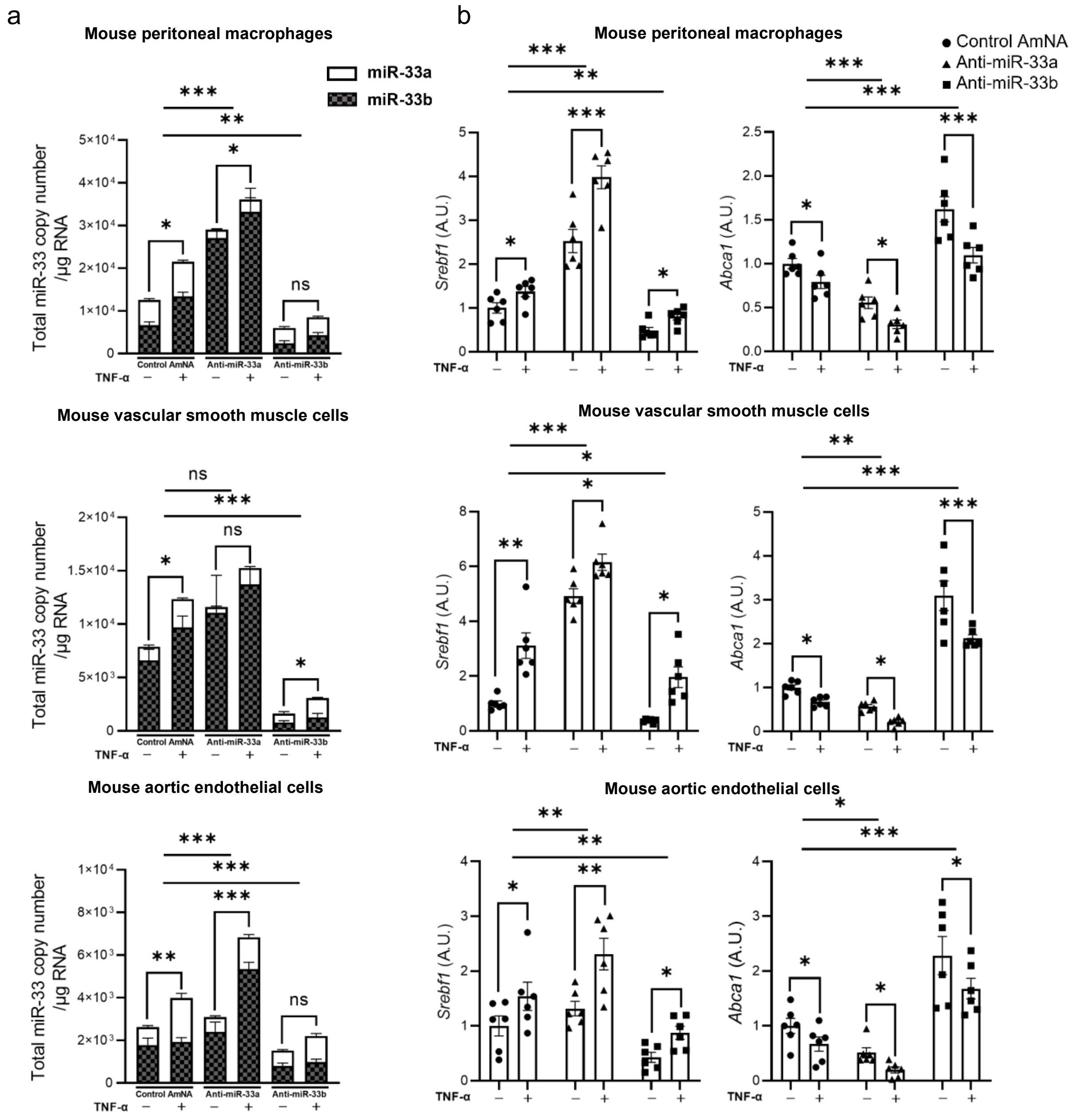

(a) Absolute copy number of miR-33a and miR-33b in miR-33b KI mouse primary cells with or without TNF-α treatment. Two-way ANOVA with Holm-Sidak's multiple comparisons test. *P<0.05, **P<0.01 and ***P<0.001. ns is defined as not significant.

(b) Expression levels of *Srebf1* and *Abca1* in miR-33b KI mouse primary cells with or without TNF-α treatment determined using quantitative real-time PCR. Two-way ANOVA with Holm-Sidak's multiple comparisons test. *P<0.05, **P<0.01 and ***P<0.001. All data represent mean ± SEM.

Incubation with TNF-α was as follows: 3 hours at 25 ng/mL for mouse P. Ms, 4 hours at 10 ng/mL for mouse VSMCs, and 1 hour at 50 ng/mL for mouse aortic ECs. P. Ms, VSMCs and ECs represent peritoneal macrophages, vascular smooth muscle cells and endothelial cells, respectively.

Supplementary Figure S2. Comparison of expression levels of total amount of miR-33, *ABCA1* and *SREBF1* in marginal zone and central zone of human AAA lesions.

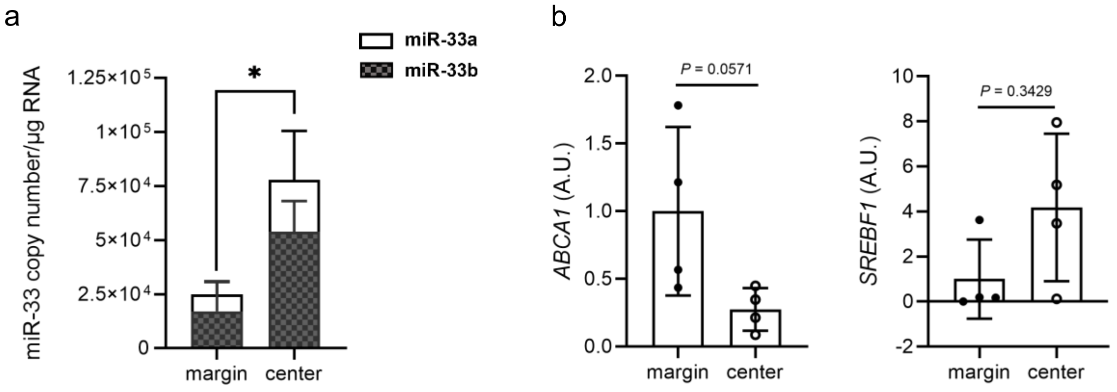

(a) Absolute copy number of miR-33a and miR-33b in marginal zone and central zone of human AAA lesions, n=4. Mann–Whitney test. *P<0.05.

(b) Expression levels of *ABCA1* and *SREBF1* in marginal zone and central zone of human AAA lesions, n=4. Mann–Whitney test. All data represent mean ± SD.

Supplementary Figure S3. Representative low power-field images of immunofluorescence staining for CD68 (red), MMP9 (green), and DAPI (blue).

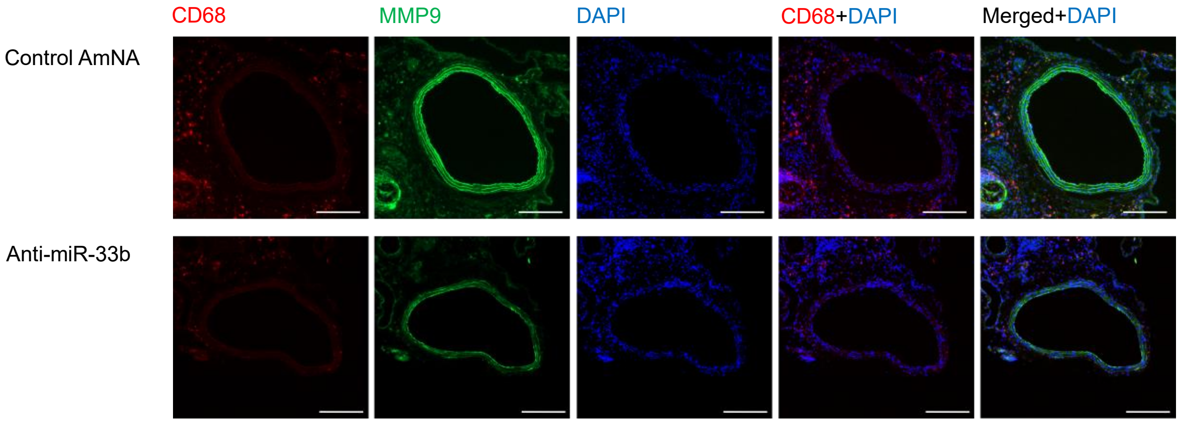

Scale bar indicates 200 μm.

Supplementary Figure S4. Scans of the original gel and blots in figure 6d and 6e.

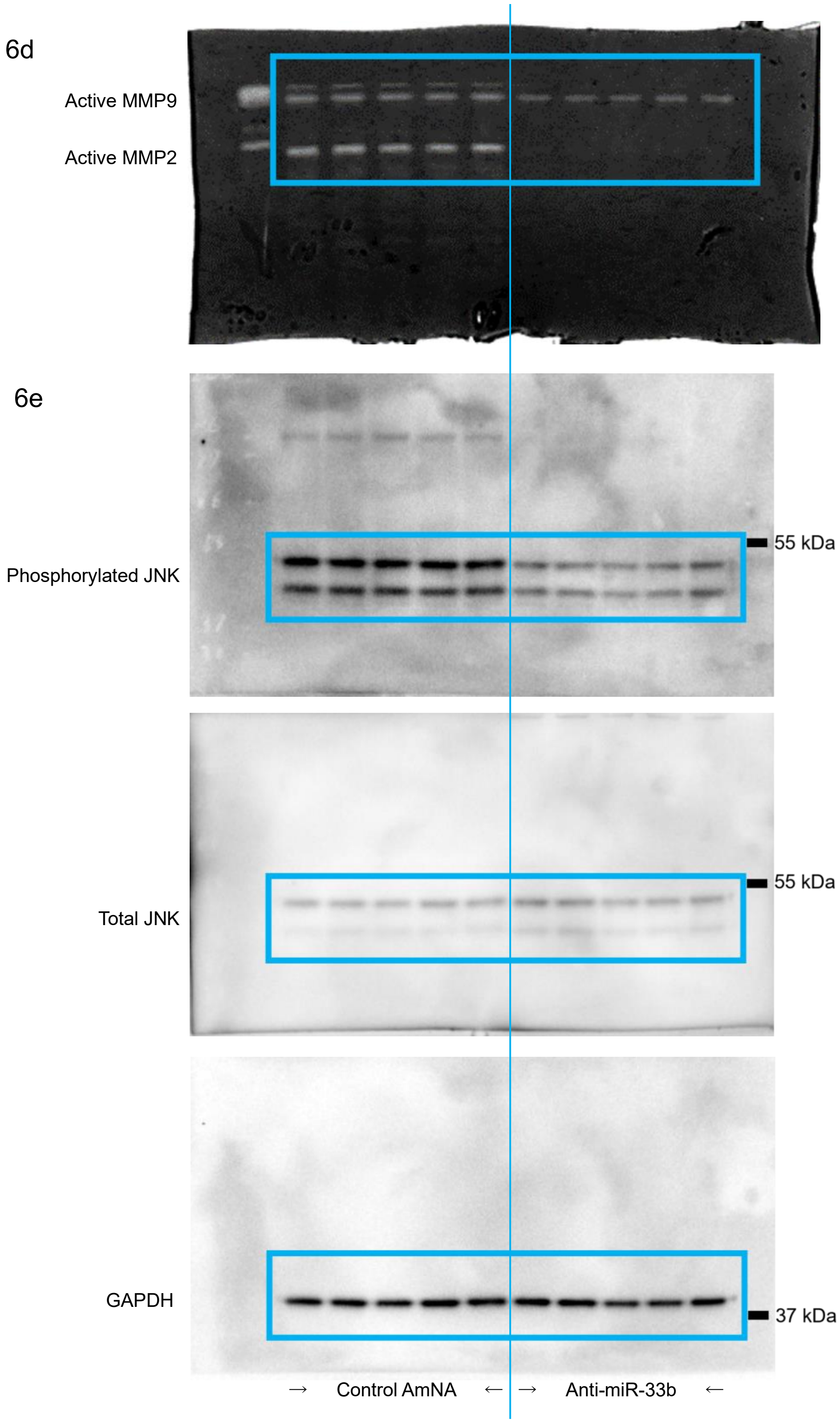

Supplementary Figure S5. Representative low power-field images of immunofluorescence staining for αSMA (red), MCP-1 (green), and DAPI (blue).

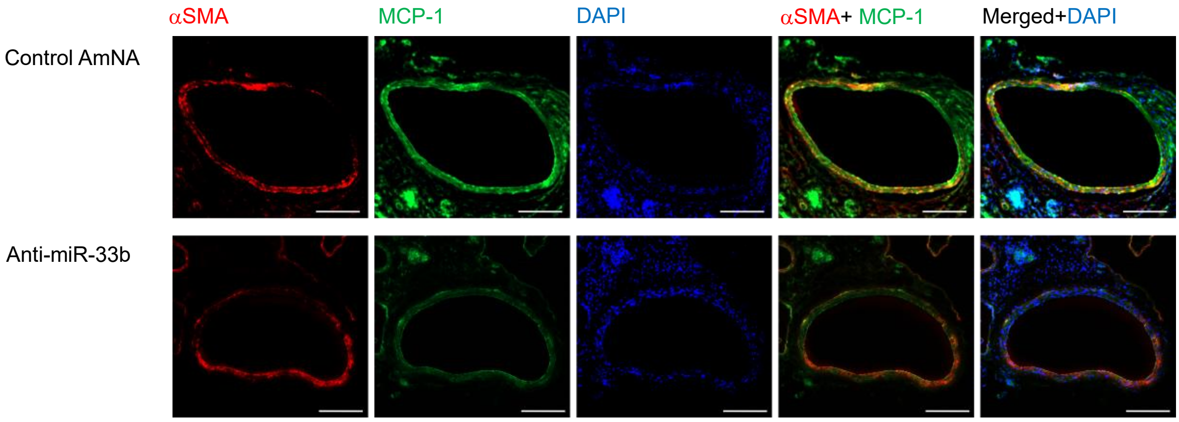

Scale bar indicates 200 μm.

Supplementary Figure S6. Validation of RNA-seq experiments by qPCR.

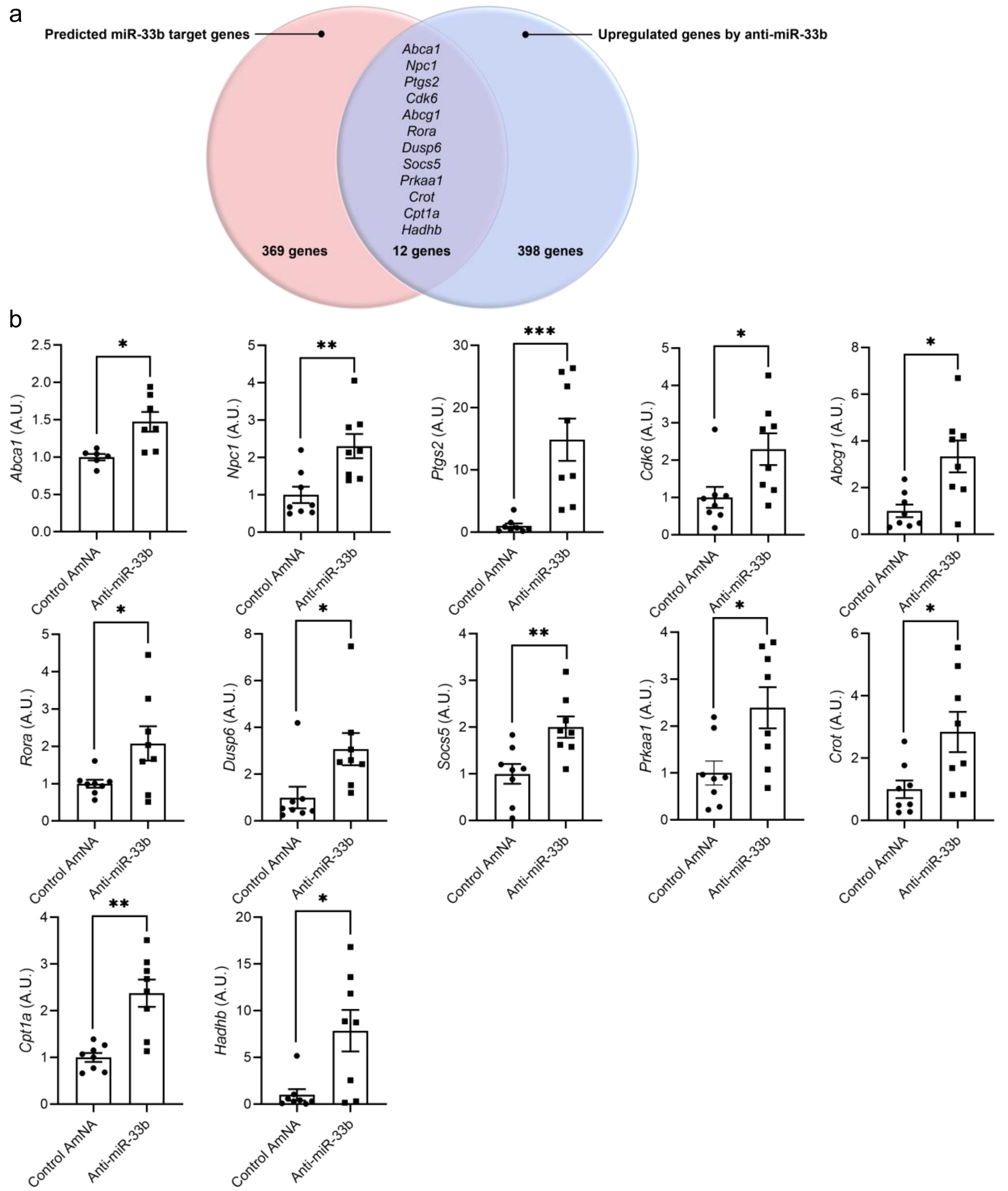

(a) Venn diagram for miR-33b target genes and upregulated genes by anti-miR-33b administration. Predicted miR-33b target genes common to human and mouse were extracted from TargetScanHuman 8.0 (https://www.targetscan.org/vert_80/).

(b) Validation of gene expression overlapped in the Venn diagram, n=8 in each group. Unpaired two-tailed t test (*Abca1*, *Rora*, *Dusp6*, *Socs5, Prkaa1* and *Crot*), unpaired t test with Welch's corresction (*Npc1*) and Mann–Whitney test (*Ptgs2*, *Cdk6*, *Abcg1*, *Cpt1a* and *Hadhb*). *P<0.05, **P<0.01 and ***P<0.001. All data represent mean ± SEM.

Supplementary Table S1. Sequences of candidates of AMOs against miR-33a and miR-33b and control oligonucleotides including AmNA.

Anti-miR-33a oligonucleotides

33a-1[12]	A(Y)^c^T(Y)^a^5(Y)^a^A(Y)^t^G(Y)^c^A(Y)^c
33a-1[14]	5(Y)^a^A(Y)^c^T(Y)^a^5(Y)^a^A(Y)^t^G(Y)^c^A(Y)^c
33a-1[16]	T(Y)^g^5(Y)^a^A(Y)^c^T(Y)^a^5(Y)^a^A(Y)^t^G(Y)^c^A(Y)^c
33a-2[12]	A(Y)^a^5(Y)^t^A(Y)^c^A(Y)^a^T(Y)^g^5(Y)^a
33a-2[14]	G(Y)^c^A(Y)^a^5(Y)^t^A(Y)^c^A(Y)^a^T(Y)^g^5(Y)^a
33a-2[16]	A(Y)^t^G(Y)^c^A(Y)^a^5(Y)^t^A(Y)^c^A(Y)^a^T(Y)^g^5(Y)^a

Anti-miR-33b oligonucleotides

33b-1[12]	A(Y)^c^A(Y)^g^5(Y)^a^A(Y)^t^G(Y)^c^A(Y)^c
33b-1[14]	5(Y)^a^A(Y)^c^A(Y)^g^5(Y)^a^A(Y)^t^G(Y)^c^A(Y)^c
33b-1[16]	T(Y)^g^5(Y)^a^A(Y)^c^A(Y)^g^5(Y)^a^A(Y)^t^G(Y)^c^A(Y)^c
33b-2[12]	A(Y)^a^5(Y)^a^G(Y)^c^A(Y)^a^T(Y)^g^5(Y)^a
33b-2[14]	G(Y)^c^A(Y)^a^5(Y)^a^G(Y)^c^A(Y)^a^T(Y)^g^5(Y)^a
33b-2[16]	A(Y)^t^G(Y)^c^A(Y)^a^5(Y)^a^G(Y)^c^A(Y)^a^T(Y)^g^5(Y)^a

Control AmNA

	A(Y)^a^5(Y)^a^A(Y)^t^A(Y)^c^T(Y)^a^5(Y)^g

Lower case, N(Y), 5(Y) and ^ indicate deoxyribonucleic acid, amido-bridged nucleic acids (AmNA), 5-methylcytosine AmNA, and phosphorothioate internucleotide linkage, respectively.

Supplementary Table S2. Used gene-specific oligonucleotide primer sequences.

Gene	Species	Forward/Reverse
Srebf1	Mouse	5'-TAGAGCATATCCCCCAGGTG-3'/5'-GGTACGGGCCACAAGAAGTA-3'
Srebf2	Mouse	5'-GTGGAGCAGTCTCAACGTCA-3'/5'-TGGTAGGTCTCACCCAGGAG-3'
Abca1	Mouse	5'-AACAGTTTGTGGCCCTTTTG-3'/5'-AGTTCCAGGCTGGGGTACTT-3'
Mmp9	Mouse	5'-TCACACGACATCTTCCAGTACC-3'/5'-CACCTCATTTTGGAAACTCACA-3'
Mcp-1	Mouse	5'-CTGGATCGGAACCAAATGAG-3'/5'-TGAGGTGGTTGTGGAAAAGG-3'
Jnk1	Mouse	5'-GCTGTGTGGAATCAAGCACC-3'/5'-AGCGAGTCACCACATAAGGC-3'
Tnfa	Mouse	5'-CCAGACCCTCACACTCAGATC-3'/5'-CACTTGGTGGTTTGCTACGAC-3'
Il1b	Mouse	5'-TCAGGCAGGCAGTATCACTCA-3'/5'-GGAAGGTCCACGGGAAAGAC-3'
Il6	Mouse	5'-ACCACGGCCTTCCCTACTTC-3'/5'-AGATTGTTTCTGCAAGTGCATCA-3'
Arg1	Mouse	5'-AACTCTTGGGAAGACAGCAGAG-3'/5'-GTAGTCAGTCCCTGGCTTATGG-3'
Crot	Mouse	5'-TACTTTTACCACGGCCGAAC-3'/5'-GACGGTCAAATCCTTTTCCA-3'
Prkaa1	Mouse	5'-AGAGGGCCGCAATAAAAGAT-3'/5'-TGTTGTACAGGCAGCTGAGG-3'
Npc1	Mouse	5'-ACTCTTGTGTGGGAGGGATG-3'/5'-AGCAGTCCTGGCAGCTACAT-3'
Ptgs2	Mouse	5'- GCGAGCTAAGAGCTTCAGGA-3'/5'- TCATACATTCCCCACGGTTT-3'
Pck1	Mouse	5'-CTGGCACCTCAGTGAAGACA-3'/5'-TCGATGCCTTCCCAGTAAAC-3'
Cpt1a	Mouse	5'-GATCTACAATTCCCCTCTGCTCT-3'/5'-TAGAGCCAGACCTTGAAGTAACG-3'
Ppargc1a	Mouse	5'-ATCACGTTCAAGGTCACCCTAC-3'/5'-GCTTCTGCCTCTCTCTCTGTTT-3'
Abcg1	Mouse	5'-GTACCATGACATCGCTGGTG-3'/5'-AGCCGTAGATGGACAGGATG-3'
Hadhb	Mouse	5'-GAGCTGTTCTTCCCAACTGC-3'/5'-AACCCGAAAGTGCAGCTCTA-3'
Rora	Mouse	5'-ACGCCCACCTACAACATCTC-3'/5'-TCACATATGGGTTCGGGTTT-3'
Dusp6	Mouse	5'-GAATAATCCGGCGAGAAACA-3'/5'-AATGAAGGTGCCCAGTTTTG-3'
Socs5	Mouse	5'-GTAAAAGCCCCAGTGTGCAT-3'/5'-CTGGGCATCTCCTAGTCTCG-3'
Aldh1a2	Mouse	5'-TTGCAGATGCTGACTTGGAC-3'/5'-TCTGAGGACCCTGCTCAGTT-3'
Pdk4	Mouse	5'-GCCTTGGGAGAAATGTGTGT-3'/5'-GAAGGCACTGGCTTTTTGAG-3'
Cdk6	Mouse	5'-TGTTTCAGCTTCTCCGAGGT-3'/5'-CTGGACTGGAGCAGGACTTC-3'
Rn18s	Mouse	5'-CGCGGTTCTATTTTGTTGGT-3'/5'-AGTCGGCATCGTTTATGGTC-3'
SREBF1	Human	5'-AACAGTCCCACTGGTCGTAGAT-3'/5'-TGTTGCAGAAAGCGAATGTAGT-3'
ABCA1	Human	5'-GTCCTCCCGCATTATCTGG-3'/5'-AGTTCCTGGAAGGTCTTGTTCAC-3'
ACTB	Human	5'-AGGCACTCTTCCAGCCTTCC-3'/5'-GCACTGTGTTGGCGTACAGG-3'
